# Novel 6a,12b-Dihydro-6*H*,7*H*-chromeno[3,4-c] chromen-6-ones: Synthesis, Structure and Antifungal Activity

**DOI:** 10.3390/molecules24091745

**Published:** 2019-05-05

**Authors:** Jin-ping Bao, Cui-lian Xu, Guo-yu Yang, Cai-xia Wang, Xin Zheng, Xin-xin Yuan

**Affiliations:** 1 College of Resource and Environment; Henan Agricultural University, Zhengzhou 450002, China; baojinping666@126.com (J.-p.B.); 18339992125@163.com (X.-x.Y.); 2 School of Science, Henan Agricultural University, Zhengzhou 450002, China; wcx670815@163.com (C.-x.W.); zhengxin@henau.edu.cn (X.Z.)

**Keywords:** dihydrocoumarins, synthesis, 3-trifluoroacetyl coumarins, phenols, antifungal activities

## Abstract

A new series of coumarin derivatives, 7-hydroxy-7-(trifluoromethyl)-6a,12b-dihydro-6*H*,7*H*-chromeno[3,4-c]chromen-6-ones **3a**–**p**, were synthesized via Michael addition, transesterification and nucleophilic addition from the reaction of 3-trifluoroacetyl coumarins and phenols in the presence of an organic base. The products were characterized by infrared spectroscopy (IR), hydrogen nuclear magnetic resonance spectroscopy (^1^H-NMR), carbon nuclear magnetic resonance spectroscopy (^13^C-NMR) and high-resolution mass spectrometer (HRMS). Single crystal X-ray analysis of compounds **3a** and **3n** clearly confirmed their assigned chemical structures and their twisted conformations. Compound **3a** crystallized in the orthorhombic system, Pbca, in which a = 8.6244(2) Å, b = 17.4245(4) Å, c = 22.5188(6) Å, α = 90°, β = 90°, γ = 90°, v = 3384.02(14) Å^3^, and z = 8. In addition, the mycelial growth rate method was used to examine the in vitro antifungal activities of the title compounds **3a**–**p** against *Fusarium graminearum* and *Fusarium monitiforme* at 500 µg/mL. The results showed that compound **3l** exhibited significant anti-*Fusarium monitiforme* activity with inhibitory index of 84.6%.

## 1. Introduction

Many natural compounds, such as coumarins, have commonly been used as meaningful lead compounds in the founding of some newer pharmaceuticals [1,2]. Coumarins belong to a class of notable heterocycle compounds containing oxygen, which exhibit various biological activities, including antioxidant [3], antitubercular [4], antitumor [5], antifungal [6], antibacterial [7], antiviral [8], antileishmanial [9], and anticancer [10] activities.

4-Arylcoumarins (neoflavones), present in many natural compounds, are considered important privileged structures due to their diverse biological activities, such as anticancer [11], antioxidant [12], antimicrobial [13], antiprotozoal [14] and antifungal activities [15]. Many studies have focused on the synthetic methods and structure-activity relationship research of 4-arylcoumarins, expecting to discover novel lead compounds [16].

Recently, heterocyclic fused coumarin derivatives from natural or non-natural products have attracted great interest from chemists and pharmaceutical scientists. For instance, pyrano-fused coumarin derivatives have shown much higher antifungal activities [17], and compounds containing thieno[3,2-c]coumarin and pyrazolo[4,3-c]coumarin frameworks demonstrated considerable antifungal and antibacterial activities in the in vitro test systems [18].

The attachment of a fluorine atom or fluorine-containing functional groups to heterocycle molecules often results in an increase in their lipophilicity, metabolic stability and bioactivity [19]. Trifluoromethyl ketones can be used as a typical structural framework to incorporate CF_3_ in the target compounds [20,21,22,23]. Keeping these aspects in mind, we adopted 3-trifluoroacetyl coumarins as a building block to synthesize novel coumarins containing fluorine [24].

Here, we report the unexpected synthesis of a series of novel dihydrocoumarins or benzopyran fused dihydrocoumarins, chromeno [3,4-c]chromen-6-ones and the preliminary evaluation of their antifungal activities.

## 2. Results and Discussion

### 2.1. Chemistry

**Scheme 1:**
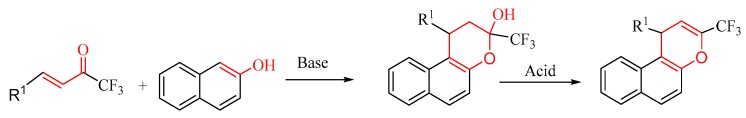
A base-catalyzed cycloaddition reaction of unsaturated ketones with phenols.

Fan et al. reported a tandem reaction of α,β-unsaturated trifluoromethyl ketones with 2-naphthol to obtain the corresponding benzo[f]chromene derivatives in the presence of catalysts with a one-pot reaction (Scheme 1) [25]. According to the literature, an 1,4-addition reaction should take place between α,β-unsaturated trifluoromethyl ketones and 2-naphthols under the catalysis of a weak base.

**Scheme 2:**
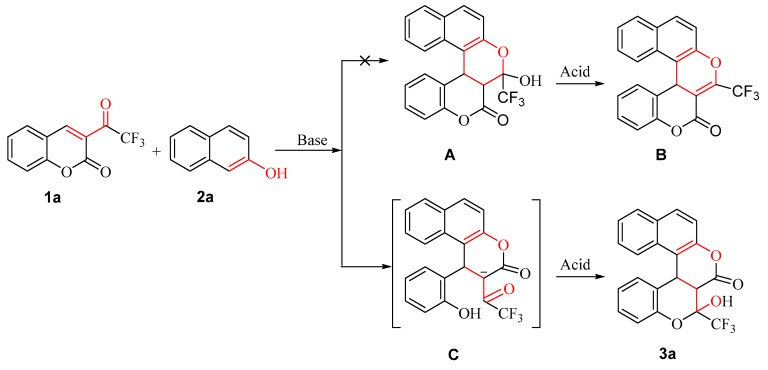
Synthesis of 3-(trifluoroacetyl)coumarin with 2-naphthol.

Inspired by Fan’s work, we anticipated synthesizing 3-hydroxy-3-(trifluoromethyl)-2a,10c-dihydro-2*H*,3*H*-benzo[f]chromeno[3,4-c]chromen-2-one (**A**) first and then 3-(trifluoromethyl)-2*H*,10c*H*-benzo[f]chromeno[3,4-c]chromen-2-one (**B**) by using coumarins containing CF_3_ (**1a**) and 2-naphthol as starting materials in a preliminary experiment (Scheme 2). However, we did not obtain the expected products (**A** and **B**). Two new dihydrocoumarin derivatives, 1-(2-hydroxyphenyl)-3-oxo-2-(2,2,2-trifluoroacetyl)-2,3-dihydro-1H-benzo[f]chromen-2-ide (**C**) and 2-hydroxy-2-(trifluoromethyl)-2a,10c-dihydro-2H,3H-benzo[f]chromeno[3,4-c]chromen-3-one (**3a**), were unexpectedly synthesized instead.

Dihydrocoumarin is an important core structure of many bioactive compounds. For example, the derivatives of dihydrocoumarin have dual physiological activities in plants and various pharmacological effects on the human body, such as antioxidant, antiviral and antibacterial effects [26,27]. Therefore, it is necessary to explore whether this reaction can be developed into a new method for the preparation of dihydrocoumarin derivatives.

We initiated the model reaction of 3-(trifluoroacetyl)coumarin (**1a**) with 2-naphthol (**2a**) to establish optimal reaction conditions. The results of the optimization were presented in Table 1.

The reaction proceeded satisfactorily at 45 °C in dichloromethane (DCM) using *N*,*N*-diisopropylethylamine (*i*-Pr_2_NEt) or triethylamine (NEt_3_) as a catalyst, resulting in a yield of 85% (Table 1, Entries 1 and 2). If the base was changed to a strong base, such as 1,8-diazabicyclo[5.4.0]undec-7-ene (DBU), the yield slightly decreased (80%) (Table 1, Entry 3). None of the other bases, such as dimethylaminopyridine (DMAP), *N*-methyl imidazole (NMI), Py, *N*-methylmorpholine (NMM), tetramethylethylenediamine (TMEDA), 1,4-diazabicyclooctane triethylenediamine (DABCO) and inorganic bases, could produce good yields (Table 1, Entries 4–12). For acidifiers, the results showed that the best yield of 85% was obtained in the presence of hydrochloric acid or concentrated sulfuric acid (Table 1, Entries 2 and 13).

Further experiment indicated that this reaction was sensitive to solvents. The yield was compared to that in DCM when being carried out in CH_3_CN, but dropped significantly when in 1,2-dichloroethane (1,2-DCE), tetrahydrofuran (THF), CHCl_3_ and dioxane (Table 1, Entries 17–20). Moreover, increasing or lowering the reaction temperature resulted in a decreased yield (Table 1, Entries 21–23) and 45 °C was identified as the ideal reaction temperature for this reaction. Gratifyingly, when the mole ratio of **2a**, **1a** and NEt_3_ was changed from 1:1:1 to 1:1.2:1.4, the reaction gave the best yield (93%) of **3a** (Table 1, Entries 24–29). Further increasing the ratio of **2a**, **1a** and NEt_3_ failed to provide a better result.

**Scheme 3:**
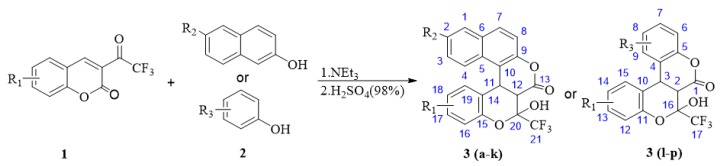
Synthesis of compounds through reaction of **1** and **2**.

Sixteen coumarin derivatives, 6a,12b-dihydro-6*H*,7*H*-chromeno[3,4-c] chromen-6-ones, were prepared from 3-(trifluoroacetyl)coumarin (1) and phenols or 2-naphthol (2), in the presence of NEt_3_ as a catalyst and hydrochloric acid as an acidifier (Scheme 3 and Table 2).

As seen from Table 2, a variety of coumarins bearing either electron-withdrawing or electron-donating groups on the aryl ring could be transformed into the corresponding novel dihydrocoumarin compounds with CF_3_, **3a–p**, with good to excellent yields (65–94%) by this simple procedure (Scheme 3). The reactions between coumarins **1** and 2-naphthols proceeded smoothly in most cases to give the products **3a**–**k** at a relatively lower temperature (45 °C) in a short time (0.3–2 h) (Entries 1–11). However, the reactions of coumarins **1** with phenols required a higher temperature (65 °C) and longer time (3–6 h) for a lower yield, below 90% (Entries 13–16). From this point of view, the electronic density of 2-naphthols was crucial for this reaction, and the electron-donating group on the naphthalene ring was favorable to the reaction rate and yield. Indeed, the yields decreased slightly in the case of strong electron-withdrawing groups, such as CN, on the naphthalene ring, and a slightly longer reaction time of 2 h was needed (Entries 10 and 11). The results were similar for the reactions between coumarins **1** and phenols. It was not until there were strong electron-donating groups, such as OH, on the phenol ring that the reactions could run smoothly at a higher temperature (65 °C) and longer times of 3–6 h (Entries 12–16).

### 2.2. Structural Characterization of Chromenes ***3a**–**p***

The structures of the synthesized compounds were confirmed by ^1^H-NMR, ^13^C-NMR, IR, and HRMS. For example, the ^1^H-NMR spectrum of **3a** showed two doublet signals for CH at 3.72 ppm and 5.26 ppm, respectively, a broad singlet signal for OH at 7.48 ppm and a multiplet signal at 6.59–8.06 ppm for aromatic protons. The downfield shift in the signal of two protons at the 3- and 4-positions, which appeared at 3.0 ppm and 4.2 ppm, respectively, in reported 3,4-dihydrocoumarins [13], was caused by the inductive effects of neighboring OH and CF_3_ groups. The decoupled ^13^C-NMR spectrum of **3a** showed 17 distinct resonance structures, in agreement with the proposed structure. Among them, the two quartet signals for C-CF_3_ at 95.71 ppm and CF_3_ at 122.13 ppm for which ^2^*J*_C,F_ = 33 Hz and ^1^*J*_C,F_ = 286 Hz, respectively, were observed. The IR spectrum of **3a** displayed characteristic OH, C=O and C-F vibrations at 3419, 1728 and 822 cm^–1^, respectively. The HRMS data for **3a** showed the molecular ion peak at *m/z* [M − H]^+^ was 385.0688, consistent with the calculated value of 385.0688.

**Figure 1:**
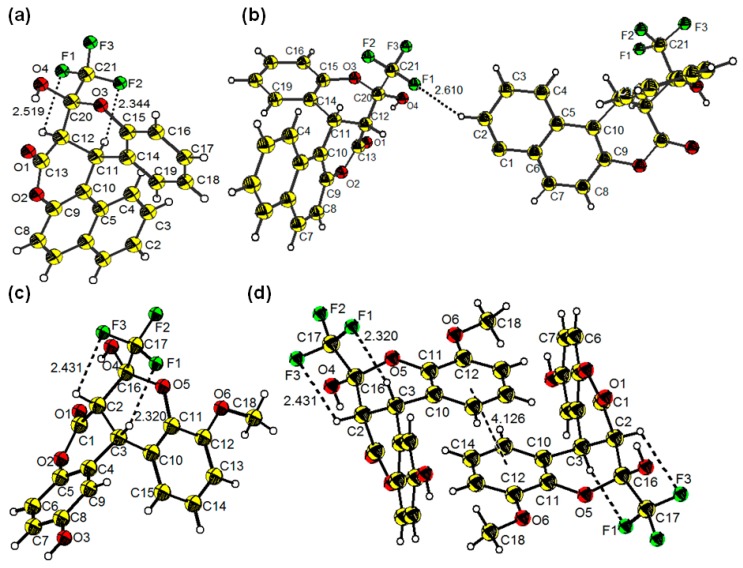
Thermal ellipsoid (30%) drawings of compound **3a** (**a**); packing of the crystal structure of **3a** (**b**); thermal ellipsoid (30%) drawings of compound **3n** (**c**); and intermolecular hydrogen bonding of compound **3n** (**d**). Note: yellow, carbon; white, hydrogen; red, oxygen; green, fluorine.

By cooling the solutions of **3a** and **3n** in ethyl acetate, single crystals suitable for X-ray crystallographic analysis were obtained, and their crystal structures are shown in Figure 1. Compound **3a** has a twisted conformation. Its benzene ring and naphthalene ring are connected by the sp3 carbon atom of C11 with a C10–C11–C14 angle of 115.01(16)°, and a dihedral angle of 72.776° between the benzene ring and the naphthalene ring is observed. Interestingly, the rotation of the chiral carbon atoms of C11 and C12 are restricted by intramolecular hydrogen bonding between H on C12 and C11 and F in trifluoromethyl groups, with CH···F distances of 2.519 and 2.344 Å, respectively. Moreover, the adjacent molecules are connected by intermolecular hydrogen bonding with a distance of 2.610 Å, which is almost equal to the van der Waals radius (2.6 Å) [28]. In comparison to **3a**, **3n** also has a twisted conformation. However, two benzene rings are almost perpendicular to each other with a dihedral angle of 89.045°, and the C4–C3–C10 angle of 113.38(16)° is slightly narrowed in comparison to that in **3a**. In addition, **3n** shows weak intermolecular interactions with a centroid distance of 4.126 Å between adjacent methoxy-substituted benzene rings (Figure 1d). Both compounds **3a** and **3n** exhibit three chiral carbon atoms, C11, C12, and C21 and C2, C3, and C16, respectively. The refinement information of compounds **3a** (C_21_H_13_F_3_O_4_) and **3n** (C_18_H_13_F_3_O_6_) is summarized in the Supplementary Materials. 

### 2.3. Reaction Mechanism

**Figure 2:**
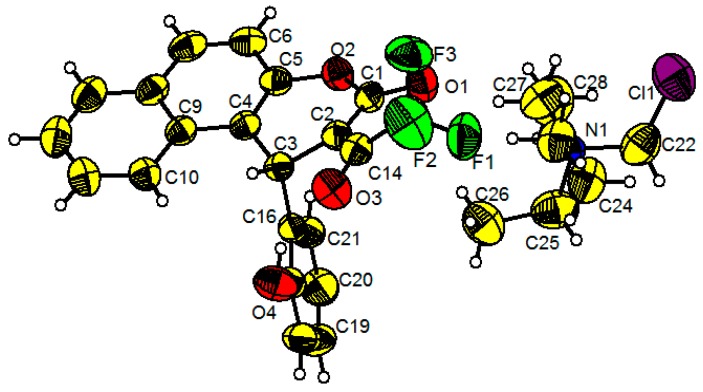
The ammonium salt **3a^1^** of compound **3a** (CCDC 1900315). Note: yellow, carbon; white, hydrogen; red, oxygen; green, fluorine; blue, nitrogen; purple, chlorine.

To speculate on the mechanism of the reaction, we obtained the crystals of compound **3a** before acidification, and it was ammonium salt **3a^1^** of compound **3a**, being formed from the reaction between Et_3_N and DCM (Figure 2). Compound **3a** exists in an open form with a free carbonyl group attached to -CF_3_ in the presence of base and easily forms a semiacetal structure with a stable six-membered ring by internal nucleophilic attack of the OH group to an active C=O group after acidification.

**Scheme 4:**
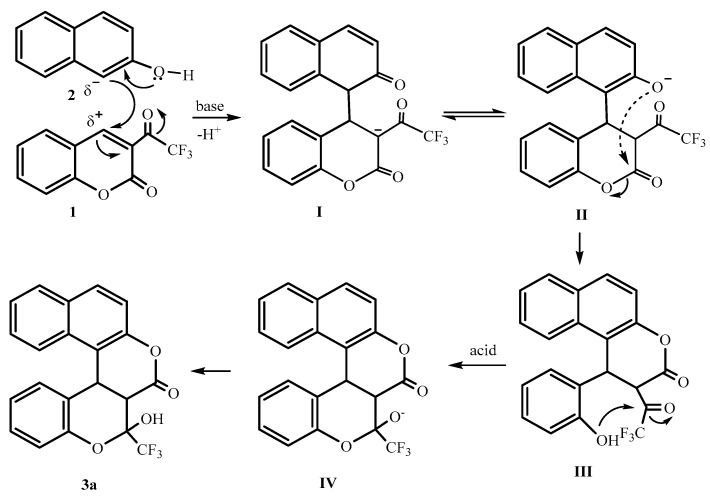
Plausible mechanism for compound **3a.**

Scheme 4 shows plausible pathways for the reaction. First, the 1,4-nucleophilic addition of 2-naphthol (**2**) to 3-trifluoroacetyl coumarin **1** with an α,β-unsaturated ketone structure generates the carbon anion intermediate **I.** It then forms enolate anion **II** by H-transformation. Intermediate **II** is next transformed into a new coumarin with phenoxy anion **III** through nucleophilic attack by a naphthalenyl oxyanion at the initial ester group of coumarin with slightly stronger basicity as the driving force. The subsequent addition of a phenoxy anion to the electrophilic carbon of -COCF_3_ group happened to furnish annulation product **IV**, forming the final title compound **3a** with a semiacetal structure after acidification.

### 2.4. In Vitro Antifungal Assay

**Figure 3:**
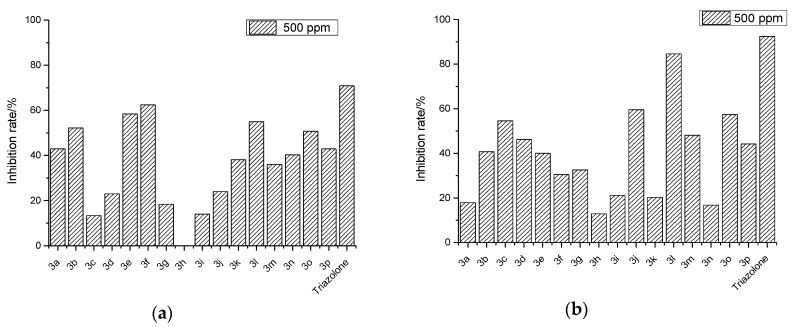
Antifungal activity of the target compounds 3**a**–**p**, against—*Fusarium graminearum* (**a**) and *Fusarium monitiforme* (**b**).

The antifungal activities of the target compounds **3a**–**p** were evaluated against *Fusarium graminearum* and *Fusarium monitiforme* by the mycelial growth rate method [29] at 500 µg/mL, using triazolone (100 µg/mL) as the positive control. The results of the antifungal activities were shown in Figure 3.

The bioassay results for *Fusarium graminearum* indicated that, except for compound **3h,** all the target compounds showed antifungal activities to some extent, with an inhibitory index in the range of 13.4–62.4%. The inhibitory index of compounds **3a** and **3l** were 42.9% and 55%, respectively. From the activity results for the derivatives (**3b**–**k**) of compound **3a** obtained by structure modification, compared with compound **3a**, compounds **3b**, **3e** and **3f** showed greater inhibition of *F. graminearum,* with inhibitory indexes of 52.2%, 58.4% and 62.4%, respectively.

The obtained findings suggested that a functional group, such as bromine, introduced into compound **3a** could substantially contribute to the antifungal efficacy of its derivatives. However, the derivatives (**3m**–**p**) of compound **3l,** whether bearing electron-donating or electron-withdrawing groups, all showed lower antifungal activities against *F. graminearum* than that of compound **3l**.

Moreover, the inhibitory index of compound **3l** against *F. monitiforme* (84.6%) was much higher than that of compound **3a** (17.9%), which indicated that compared with the benzene ring, the naphthalene ring at the 4-position of dihydrocoumarin is unfavorable for activity against *F. monitiforme.* However, the compounds **3b**–**k** modified from compound **3a**, except compound **3h**, all had higher antifungal activities against *F. monitiforme* (inhibitory index: 21.2–59.6%) than compound **3a**. The reason may be that the introduction of a functional group can increase the antifungal activity. Similar to the antifungal results against *F. graminearum,* the compounds (**3m**–**p)** modified from compound **3l** all showed lower antifungal activities against *F. monitiforme* (inhibitory index: 16.8%–57.4%) than compound **3l**. Further research about structure and activity is in progress.

## 3. Materials and Methods

### 3.1. General Information

HPLC analyses were performed with Thermo Fisher U-3000 (Chromatographic column: XDB-C_18_ column with MeOH-H_2_O as the eluent, (Thermo Fisher scientific Co., Waltham, MA, USA) equipped with an UltiMate 3000UV detector (Thermo Fisher scientific Co., Waltham, MA, USA). Melting points were determined on an X-5 digital microscopic melting-point apparatus (Beijing Tech Instruments Co., Beijing, China) and were uncorrected. High resolution mass spectra were obtained using a Waters Q-Tof MicroTM instrument (Waters Co., MA, USA). NMR spectra were recorded on a Bruker DPX-400 spectrometer (Bruker Optics Co., Ettlingen, baden-wuerttemberg, Germany) in dimethyl sulfoxide- *d*_6_ (DMSO-*d*_6_) or chloroform-D (CDCl_3_) using tetramethylsilane as the internal standard. Chemical shifts (δ) were reported in ppm, and *J* values were reported in Hertz. The IR spectra were recorded on a Thermo IS10 FT-IR spectrometer (Thermo scientific Co., Shenzhen, Guangdong, China), and the frequencies were reported in cm^−1^. X-ray images were obtained with a Rigaku RAXIAS-IV type diffractometer (Rigaku Corporation Co., Tokyo, Japan).

The starting materials (**1**) were obtained according to the reported procedure [30]. All other reagents were acquired from commercial sources and utilized without further purification.

### 3.2. General Synthetic Procedures for Compounds ***3a**–**p***

Typical procedure for the synthesis of compound **3a**: compound **1** (0.64 mmol), naphthol (0.50 mmol), NEt_3_ (0.71 mmol) and 5 mL CH_3_CN were added to a pressure vial. The reaction mixture was stirred at 45 °C for 1.5 h (HPLC tracking reaction), and then cooled down to room temperature. After the addition of concentrated H_2_SO_4_ (0.71 mmol), the resulting mixture was stirred at room temperature for 2 h. The crude reaction mixture was concentrated by rotary evaporator, and dissoloved in DCM 30 mL. Then it was washed with water (15 mL × 3) and the organic layers were dried with Na_2_SO_4_, filtered, and concentrated in vacuo. A white crystal was obtained after crystallization from ethyl acetate-petroleum ether (0.183 g, 93%).

### 3.3. Biological Assays

The antifungal activity of synthesized compounds **3a**–**p** was tested against two pathogenic fungi, namely, *Fusarium graminearum* and *Fusarium monitiforme*, by the poison plate technique at a concentration of 500 µg/mL. The two species of fungi were incubated in potato dextrose agar medium at 25 ± 1 °C for five days to obtain new mycelia for the antifungal assay, and then mycelia as disks of approximately 0.70 cm diameter cut from the culture medium were picked up with a sterilized inoculation needle and inoculated into the center of a PDA plate. The test compounds were dissolved in dimethyl sulfoxide (1 mL) then diluted to a 7500 µg/mL drug solution with 4% tween-80 emulsifier (1 mL) and added to the PDA medium (30 mL). The final concentration of compounds in the medium was adjusted to 500 µg/mL. The inoculated plates were incubated at 25 ± 1 °C for 3 days. Dimethyl sulfoxide was diluted with sterilized distilled water (4% tween-80) and used as the control, while the commercial fungicide triazolone (100 µg/mL) was used as the standard control. Three replicates of the experiments were performed. The radial growth of the fungal colonies was measured on the fourth day.

*2-Hydroxy-2-(trifluoromethyl)-2a,10c-dihydro-2H,3H-benzo[f]chromeno[3,4-c]chromen-3-one* (**3a**): Crystallization from ethyl acetate-petroleum ether, white crystal; M.p. 172.6~173.7 °C; ^1^H-NMR (CDCl_3_, 400 MHz) δ: 3.72 (d, *J* = 4 Hz, 1H, CH), 5.26 (d, *J* = 4 Hz, 1H, CH), 6.59 (d, *J* = 4 Hz, 1H, Ar-H), 6.70 (td, *J*_1_ = 8 Hz, *J*_2_ = 1.2 Hz, Ar-H), 7.12 (q, *J* = 4 Hz, 1H, Ar-H), 7.23~7.33 (m, 2H, Ar-H), 7.48 (s, 1H, -OH), 7.64 (td, *J*_1_ = 8 Hz, *J*_2_ = 0.8 Hz, 1H, Ar-H), 7.74 (td, *J*_1_ = 8 Hz, *J*_2_ = 1.2 Hz, 2H, Ar-H), 7.98~8.06 (m, 3H, Ar-H); ^13^C-NMR (100 MHz, CDCl_3_) δ: 31.12 (q, *J* = 3 Hz, C1), 39.46 (C1), 95.71 (q, *J* = 33 Hz, C20), 116.24 (C16), 116.94 (C18), 117.60 (C8), 118.29 (C10), 122.13 (q, *J* = 286 Hz, C21), 122.64 (C4), 122.90 (C2), 126.20 (C3), 127.61 (C17), 128.61 (C7), 129.13 (C1), 129.92 (C19), 130.88 (C6), 131.33 (C14), 131.82 (C5), 147.44 (C9), 152.64 (C15), 168.35 (C13); IR (KBr) ν_max_ (cm^−1^): 3419 (OH), 1728 (C=O), 1585 (Ar), 822 (CF_3_); HRMS (ESI): *m/z* calcd for C_21_H_12_F_3_O_4_ [M − H]^+^: 385.0688; found: 385.0688.

*2-Hydroxy-14-methoxy-2-(trifluoromethyl)-2a,10c-dihydro-2H,3H-benzo[f]chromeno[3,4-c]chromen-3-one* (**3b**): Crystallization from ethyl acetate-petroleum ether, white crystal; M.p. 173.7~174.5 °C; ^1^H-NMR (CDCl_3_, 400 MHz) δ: 3.67 (d, *J* = 4 Hz, 1H, CH), 3.88 (s, 3H, CH_3_), 5.22 (d, *J* = 4 Hz, 1H, CH), 6.13 (d, *J* = 4 Hz, 1H, Ar-H), 6.68 (t, *J* = 8 Hz, 1H, Ar-H), 6.81 (d, *J* = 8 Hz, 1H, Ar-H), 7.27 (d, *J* = 4 Hz, 1H, Ar-H), 7.47 (s, 1H, -OH), 7.58 (td, *J*_1_ = 8 Hz, *J*_2_ =0.8 Hz, 1H, Ar-H), 7.68 (td, *J*_1_ = 8 Hz, *J*_2_ = 0.8 Hz, 1H, Ar-H), 7.92~8.00 (m, 3H, Ar-H); ^13^C-NMR (100 MHz, CDCl_3_) δ: 31.16 (q, *J* = 2 Hz, C11), 39.38 (C12), 56.30 (-OCH_3_), 95.97 (q, *J* = 26 Hz, C20), 112.34 (C17), 116.29 (C19), 116.87 (C18), 118.92 (C8), 119.40 (C10), 122.08 (q, *J* = 229 Hz, C21), 122.20 (C4), 122.90 (C2), 126.10 (C3), 128.49 (C7), 129.04 (C1), 130.79 (C6),, 131.27 (C14), 131.73 (C5), 142.31 (C16), 147.41 (C15), 148.65 (C9), 168.26 (C13); IR (KBr) ν_max_ (cm^−1^): 3391 (OH), 1736 (C=O), 1582, 1478 (Ar), 817 (CF_3_); HRMS (ESI): *m/z* calcd for C_22_H_15_F_3_O_5_ [M − H]^+^: 415.0793; found: 415.0795.

*2-Hydroxy-13-methoxy-2-(trifluoromethyl)-2a,10c-dihydro-2H,3H-benzo[f]chromeno[3,4-c]chromen-3-one* (**3c**): Crystallization from ethyl acetate-petroleum ether, white crystal; M.p. 173.6~174.3 °C; ^1^H-NMR (CDCl_3_, 400 MHz) δ: 3.64 (d, *J* = 4 Hz, 1H, CH), 3.72 (s, 3H, CH_3_), 5.14 (d, *J* = 4 Hz, 1H, CH), 6.31 (dd, *J*_1_ = 8 Hz, *J*_2_ = 2 Hz, 1H, Ar-H), 6.42 (dd, *J*_1_ = 4 Hz, *J*_2_ = 0.8 Hz, 1H, Ar-H), 6.62 (d, *J* = 4 Hz, 1H, Ar-H), 7.69 (td, *J*_1_ = 8 Hz, *J*_2_ = 0.8 Hz, 1H, Ar-H), 7.92~8.00 (m, 3H, Ar-H); ^13^C-NMR (100 MHz, CDCl_3_) δ: 30.59 (q, *J* = 3 Hz, C11), 39.64 (C12), 55.39 (-OCH_3_), 95.79 (q, *J* = 26 Hz, C20), 102.11 (C16), 109.75 (C18), 110.00 (C8), 116.38 (C14), 116.90 (C10), 122.05 (q, *J* = 229 Hz, C21), 122.87 (C4), 126.11 (C2), 128.25 (C3), 128.51 (C7), 129.06 (C1), 130.64 (C19), 131.29 (C6), 131.76 (C5), 147.28 (C9), 153.55 (C15), 160.87 (C17), 168.41 (C13); IR (KBr) ν_max_ (cm^−1^): 3402 (OH), 1736 (C=O), 1628, 1501 (Ar), 819 (CF_3_); HRMS (ESI): *m/z* calcd for C_22_H_15_F_3_O_5_ [M − H]^+^: 415.0793; found: 415.0792.

*2-Hydroxy-12-methyl-2-(trifluoromethyl)-2a,10c-dihydro-2H,3H-benzo[f]chromeno[3,4-c]chromen-3-one* (**3d**): Crystallization from ethyl acetate-petroleum ether, white crystal; M.p. 160.2~161.1 °C; ^1^H-NMR (DMSO-*d*_6_, 400 MHz) δ: 1.97 (s, 3H, CH_3_), 3.61 (dd, *J*_1_ = 6 Hz, *J*_2_ = 1.2 Hz, 1H, CH), 5.60 (d, *J* = 6 Hz, 1H, CH), 6.17 (s, 1H, Ar-H), 7.02 (q, *J* = 8 Hz, 2H, Ar-H, OH), 7.25 (d, *J* = 8 Hz, 1H, Ar-H), 7.52 (t, *J* = 8 Hz, 1H, Ar-H), 7.62 (t, *J* = 8 Hz, 1H, Ar-H), 7.92 (d, *J* = 8 Hz, 1H, Ar-H), 8.00 (q, *J* = 4 Hz, 2H, Ar-H), 8.73 (d, *J* = 4 Hz, 1H, Ar-H); ^13^C-NMR (100 MHz, DMSO-*d_6_*) δ: 20.89 (-CH_3_), 29.71 (C11), 41.54 (C12), 94.85 (q, *J* = 32 Hz, C20), 112.86 (C16), 115.82 (C8), 118.63 (C10), 122.66 (q, *J* = 288 Hz, C21), 123.31 (C4), 123.43 (C2), 125.11 (C3), 126.95 (C17), 128.00 (C7), 128.85 (C1), 129.10 (C18), 129.83 (C19), 130.61 (C14), 132.80 (C6), 133.52 (C5), 148.59 (C9), 150.30 (C15) (C13), 164.50 (C=O); IR (KBr) ν_max_ (cm^−1^): 3287 (OH), 1765 (C=O), 1628, 1600 (Ar), 812 (CF_3_); HRMS (ESI): *m*/*z* calcd for C_22_H_15_F_3_O_4_ [M − H]^+^: 399.0844; found: 399.0805.

*12-Bromo-2-hydroxy-2-(trifluoromethyl)-2a,10c-dihydro-2H,3H-benzo[f]chromeno[3,4-c]chromen-3-one* (**3e**): Crystallization from ethyl acetate-petroleum ether, white crystal; M.p. 158.7~159.9 °C; ^1^H-NMR (CDCl_3_, 400 MHz) δ: 3.65 (d, *J* = 4 Hz, 1H, CH), 5.18 (d, *J* = 4 Hz, 1H, CH), 7.64 (d, *J* = 4 Hz, 1H, Ar-H), 6.97 (d, *J* = 4 Hz, 1H, Ar-H), 7.28~7.32 (m, 2H, Ar-H), 7.42 (s, 1H, -OH), 7.61 (t, *J* = 4 Hz, 1H, Ar-H), 7.72 (t, *J* = 4 Hz, 1H, Ar-H), 7.95~7.99 (m, 3H, Ar-H); ^13^C NMR (100 MHz, CDCl_3_) δ: 30.96 (C11), 39.14 (C12), 95.84 (q, *J* = 26 Hz, C20), 115.03 (C16), 115.32 (C18), 116.88 (C8), 119.44 (C10), 120.78 (C4), 121.93 (q, *J* = 229 Hz, C21), 122.47 (C2), 126.36 (C3), 128.90 (C7), 129.26 (C1), 130.11 (C17), 131.28 (C6), 131.39 (C5), 131.49 (C14), 132.99 (C19), 147.43 (C9), 151.76 (C15), 168.00 (C13); IR (KBr) ν_max_ (cm^-1^): 3408 (OH), 1740 (C=O), 1628, 1471 (Ar), 809 (CF_3_); HRMS (ESI): *m*/*z* calcd for C_21_H_11_BrF_3_O_4_ [M − H]^+^: 462.9793; found: 462.9793.

*7-Bromo-2-hydroxy-2-(trifluoromethyl)-2a,10c-dihydro-2H,3H-benzo[f]chromeno[3,4-c]chromen-3-one* (**3f**): Eluent: EA:PE:AcOH = 2:8:0.1; white crystal; M.p.185.0~186.9 °C; ^1^H-NMR (CDCl_3_, 400 MHz) δ: 3.12 (d, *J* = 6 Hz, 1H, CH), 5.19 (d, *J* = 6 Hz, 1H, CH), 6.53 (d, *J* = 8 Hz, 1H, Ar-H), 6.80 (t, *J* = 8 Hz, 1H, Ar-H), 7.11 (d, *J* = 8 Hz, 1H, Ar-H), 7.26 (t, *J* = 8 Hz, 1H, Ar-H), 7.34 (d, *J* = 2 Hz, 1H, Ar-H), 7.41 (s, 1H, -OH), 7.80 (d, *J* = 8 Hz, 1H, Ar-H), 7.88~7.92 (t, *J* = 8 Hz, 2H, Ar-H), 8.17 (s, 1H, Ar-H); ^13^C NMR (100 MHz, CDCl_3_) δ: 31.19 (d, *J* = 3 Hz, C11), 39.37 (C12), 95.65 (q, *J* = 33 Hz, C20), 116.57 (C16), 117.74 (C18), 117.93 (C1), 118.21 (C8), 120.26 (C10), 122.09 (q, *J* = 287 Hz, C21), 122.73 (C4), 124.67 (C3), 127.37 (C7), 129.93 (C17), 130.10 (C19), 130.41 (C6), 131.11 (C2), 131.94 (C14), 132.39 (C5), 147.58 (C9), 152.61 (C15), 167.96 (C13); IR (KBr) ν_max_ (cm^−1^): 3360 (OH), 1723 (C=O), 1583, 1479 (Ar), 758 (CF_3_); HRMS (ESI): *m/z* calcd for C_21_H_11_BrF_3_O_4_ [M − H]^+^: 462.9793; found: 462.9791.

*7-Bromo-2-hydroxy-14-methoxy-2-(trifluoromethyl)-2a,10c-dihydro-2H,3H-benzo[f]chromeno[3,4-c]chromen-3-one* (**3g**): Crystallization from ethyl acetate-petroleum ether, white crystal; M.p. 204.3~204.8 °C; ^1^H-NMR (CDCl_3_, 400 MHz) δ: 3.72 (d, *J* = 4 Hz, 1H, CH), 5.19 (d, *J* = 4 Hz, 1H, CH), 6.09 (d, *J* = 8 Hz, 1H, Ar-H), 7.33 (d, *J* = 8 Hz, 1H, Ar-H), 7.45 (s, 1H, -OH), 7.79 (d, *J* = 8 Hz, 1H, Ar-H), 7.89 (t, *J* = 8 Hz, 2H, Ar-H), 8.16 (s, 1H, Ar-H); ^13^C-NMR (100 MHz, CDCl_3_) δ: 31.28 (q, *J* = 3 Hz, C11), 39.33 (C12), 56.35 (-OCH_3_), 95.95 (q, *J* = 33 Hz, C20), 112.51 (C17), 116.67 (C19), 118.18 (C18), 118.69 (C1), 119.07 (C8), 120.20 (C10), 122.07 (q, *J* = 286 Hz, C21), 122.34 (C4), 124.72 (C3), 129.88 (C7), 130.37 (C6), 131.05 (C2), 131.87 (C14), 132.37 (C5), 142.33 (C16), 147.62 (C15), 148.77 (C9), 167.90 (C13); IR (KBr) ν_max_ (cm^−1^): 3416 (OH), 1762 (C=O), 1580 (Ar), 865 (CF_3_); HRMS (ESI): *m*/*z* calcd for C_22_H_14_BrF_3_O_5_ [M − H]^+^: 492.9898; found: 492.9850.

*7-Bromo-2-hydroxy-13-methoxy-2-(trifluoromethyl)-2a,10c-dihydro-2H,3H-benzo[f]chromeno[3,4-c]chromen-3-one* (**3h**): Eluent: EA:PE:AcOH = 1:8:0.25; white acicular crystal; M.p. 162.1~163.0 °C; ^1^H-NMR (CDCl_3_, 400 MHz) δ: 3.65 (d, *J* = 8 Hz, 1H, CH), 3.74 (s, 3H, CH_3_), 5.08 (d, *J* = 8 Hz, 1H, CH), 6.32~6.38 (m, 2H, Ar-H), 6.63 (d, *J* = 4 Hz, 1H, Ar-H), 7.30 (d, *J* = 8 Hz, 1H, Ar-H), 7.37 (s, 1H, -OH), 7.76 (dd, *J*_1_ = 8 Hz, *J*_2_ = 2.4 Hz, 1H, Ar-H), 7.86 (t, *J* = 8 Hz, 1H, Ar-H), 8.13 (d, *J* = 1.6 Hz, 1H, Ar-H); ^13^C NMR (100 MHz, CDCl_3_) δ: 30.68 (q, *J* = 3 Hz, C11), 39.57 (C12), 55.45 (-OCH_3_), 95.42 (q, J = 33 Hz, C20), 102.23 (C16), 109.62 (C18), 109.89 (C1), 116.74 (C8), 118.19 (C14), 120.20 (C10), 122.03 (q, *J* = 286 Hz, C21), 124.67 (C4), 128.08 (C3), 129.79 (C7), 130.37 (C6), 131.07 (C2), 131.88 (C19), 132.37 (C5), 147.48 (C9), 153.57 (C15), 161.02 (C17), 168.08 (C13); IR (KBr) ν_max_ (cm^−1^): 3419 (OH), 1737 (C=O), 1580, 1499 (Ar), 812 (CF_3_); HRMS (ESI): *m*/*z* calcd for C_22_H_14_BrF_3_O_5_ [M − H]^+^: 492.9898; found: 492.9510.

*7-Bromo-12-chloro-2-hydroxy-2-(trifluoromethyl)-2a,10c-dihydro-2H,3H-benzo[f]chromeno[3,4-c]chromen-3-one* (**3i**): Eluent: EA:PE:AcOH = 1:8:0.25; white crystal; M.p. 175.3~176.1 °C; ^1^H-NMR (CDCl_3_, 400 MHz) δ: 3.70 (d, *J* = 6 Hz, 1H, CH), 5.15 (d, *J* = 6 Hz, 1H, CH), 6.47 (s, 1H, Ar-H), 7.06 (d, *J* = 8 Hz, 1H, Ar-H), 7.22 (d, *J* = 4 Hz, 1H, Ar-H), 7.35 (d, *J* = 8 Hz, 1H, Ar-H), 7.40 (s, 1H, -OH), 7.80~7.93 (m, 3H, Ar-H), 8.18 (s, 1H, Ar-H); ^13^C-NMR (100 MHz, CDCl_3_) δ: 31.09 (d, *J* = 2 Hz, C11), 39.11 (C12), 95.83 (q, *J* = 33 Hz, C20), 115.72 (C16), 118.20 (C1), 119.21 (C8), 119.69 (C10), 120.48 (C4), 121.94 (q, *J* = 286 Hz, C21), 124.26 (C3), 127.07 (C18), 127.86 (C7), 130.12 (C17), 130.27 (C19), 130.36 (C6), 131.30 (C2), 132.26 (C14), 132.47 (C5), 147.63 (C9), 151.22 (C15), 167.64 (C13); IR (KBr) ν_max_ (cm^−1^): 3430 (OH), 1748 (C=O), 1583, 1504 (Ar), 812 (CF_3_); HRMS (ESI): *m*/*z* calcd for C_21_H_11_BrClF_3_O_4_ [M + H]^+^: 498.9560; found: 498.9331.

*2-Hydroxy-3-oxo-2-(trifluoromethyl)-2a,10c-dihydro-2H,3H-benzo[f]chromeno[3,4-c]chromene-8-carbonitrile* (**3j**): Eluent: EA:PE:AcOH = 1:4:0.25; white crystal; M.p. 174.7~175.5 °C; ^1^H-NMR (CDCl_3_, 400 MHz) δ: 3.32 (dd, *J*_1_ = 16 Hz, *J*_2_ = 2 Hz, 1H, CH), 5.34 (d, *J* = 8 Hz, 1H, CH), 5.70 (s, 1H, -OH), 6.55 (dd, *J*_1_ = 8 Hz, *J*_2_ = 0.8 Hz, 1H, Ar-H), 6.70 (t, *J* = 8 Hz, 1H, Ar-H), 6.80 (d, *J* = 4 Hz, 1H, Ar-H), 7.09 (td, *J*_1_ = 8 Hz, *J*_2_ = 1.6 Hz, 1H, Ar-H), 7.48 (d, *J* = 8 Hz, 1H, Ar-H), 7.59 (dd, *J*_1_ = 8 Hz, *J*_2_ = 1.6 Hz, 1H, Ar-H), 7.87 (d, *J* = 12 Hz, 1H, Ar-H), 7.94 (d, *J* = 12 Hz, 1H, Ar-H), 8.24 (d, *J* = 0.8 Hz, 1H, Ar-H); ^13^C-NMR (100 MHz, CDCl_3_) δ: 31.41 (C11), 35.04 (C12), 95.53 (q, *J* = 33 Hz, C20), 108.76 (C1), 115.60 (C16), 118.15 (-CN), 118.87 (C18), 119.51 (C7), 122.42 (q, *J* = 286 Hz, C21), 121.47 (C8), 124.67 (C10), 125.98 (C3), 127.84 (C17), 128.04 (C19), 129.11 (C4), 130.00 (C5), 130.35 (C6), 132.86 (C14)), 134.54 (C2), 152.40 (C15, C9), 167.22 (C13); IR (KBr) ν_max_ (cm^−1^): 3419 (OH), 2228 (CN), 1748 (C=O), 1628, 1585 (Ar), 753 (CF_3_); HRMS (ESI): *m/z* calcd for C_22_H_12_F_3_NO_4_ [M − H]^+^: 410.0640; found: 410.0600.

*2-Hydroxy-13-methoxy-3-oxo-2-(trifluoromethyl)-2a,10c-dihydro-2H,3H-benzo[f]chromeno[3,4-c]chromene-8-carbonitrile* (**3k**): Eluent: EA:PE:AcOH = 1:7:0.25; white acicular crystal; M.p. 169.7~170.4 °C; ^1^H-NMR (CDCl_3_, 400 MHz) δ: 3.68 (d, *J* = 6 Hz, 1H, CH), 3.74 (s, 3H, CH_3_), 5.13 (d, *J* = 6 Hz, 1H, CH), 5.30 (s, 1H, -OH), 6.34 (d, *J* = 4 Hz, 1H, Ar-H), 6.64 (d, *J* = 4 Hz, 1H, Ar-H), 7.25 (d, *J* = 4 Hz, 1H, Ar-H), 7.43 (d, *J* = 12 Hz, 1H, Ar-H), 7.85 (dd, *J*_1_ = 12 Hz, *J*_2_ = 2 Hz, 1H, Ar-H), 8.03 (d, *J* = 8 Hz, 1H, Ar-H), 8.11 (d, *J* = 4 Hz, 1H, Ar-H), 8.36 (s, 1H, Ar-H); ^13^C NMR (100 MHz, CDCl_3_) δ: 30.69 (q, *J* = 3 Hz, C11), 39.48 (C12), 55.47 (-OCH_3_), 95.63 (q, *J* = 30 Hz, C20), 102.34 (C16), 109.10 (C18), 109.93 (C1), 109.99 (-CN), 117.02 (C7), 118.43 (C14), 119.06 (C8), 121.96 (q, *J* = 286 Hz, C21), 124.39 (C10), 127.86 (C3), 129.08 (C4), 130.17 (C19), 131.36 (C5), 133.48 (C6), 134.92 (C2), 149.44 (C9), 153.56 (C15), 161.14 (C17), 167.55 (C13); IR (KBr) ν_max_ (cm^−1^): 3430 (OH), 2228 (CN), 1742 (C=O), 1619, 1580 (Ar), 817 (CF_3_); HRMS (ESI): *m*/*z* calcd for C_23_H_13_F_3_NO_4_ [M − H]^+^: 440.0746; found: 440.0743.

*7-Hydroxy-7-(trifluoromethyl)-6a,12b-dihydro-6H,7H-chromeno[3,4-c]chromen-6-one* (**3l**): Eluent: EA:PE:AcOH = 2:8:0.25; white acicular crystal; M.p. 151.8~152.6 °C; ^1^H-NMR (CDCl_3_, 400 MHz) δ: 3.67 (d, *J* = 6 Hz, 1H, CH), 4.59 (d, *J* = 6 Hz, 1H, CH), 6.69 (d, *J* = 8 Hz, 1H, Ar-H), 6.91 (t, *J* = 8 Hz, 1H, Ar-H), 7.07 (d, *J* = 8 Hz, 1H, Ar-H), 7.16 (d, *J* = 8 Hz, 1H, Ar-H), 7.23~7.28 (m, 1H, Ar-H), 7.38 (t, *J* = 8 Hz, 1H, Ar-H), 7.46~7.52 (m, 2H, Ar-H); ^13^C-NMR (100 MHz, CDCl_3_) δ: 34.98 (C3), 39.91 (C2), 95.50 (q, *J* = 32 Hz, C16), 117.47 (C12), 117.58 (C14), 118.41 (C6), 121.96 (C8), 122.01 (q, *J* = 287 Hz, C17), 122.54 (C13), 125.65 (C7), 127.45 (C15), 129.91 (C9), 129.99 (C10), 130.09 (C4), 149.42 (C5), 152.36 (C11), 168.11 (C1); IR (KBr) ν_max_ (cm^−1^): 3414 (OH), 1726 (C=O), 1588, 1487 (Ar), 756 (CF_3_); HRMS (ESI): *m*/*z* calcd for C_17_H_10_F_3_O_4_ [M − H]^+^: 335.0531; found: 335.0298.

*1,7-Dihydroxy-7-(trifluoromethyl)-6a,12b-dihydro-6H,7H-chromeno[3,4-c]chromen-6-one* (**3m**): Eluent: EA:PE:AcOH = 1:5:0.1; white solid; M.p. 138.5~139.3 °C; ^1^H-NMR (CDCl_3_, 400 MHz) δ: 3.60 (d, *J* = 4 Hz, 1H, CH), 4.47 (d, *J* = 4 Hz, 1H, CH), 5.12 (s, 1H, -OH), 6.61 (d, *J* = 2 Hz, 1H, Ar-H), 6.75~6.80 (m, 2H, Ar-H), 6.87 (t, *J* = 4 Hz, 1H, Ar-H), 7.01 (d, *J* = 8 Hz, 1H, Ar-H), 7.20 (t, *J* = 4 Hz, 1H, Ar-H), 7.31 (d, *J* = 8 Hz, 1H, Ar-H); ^13^C NMR (100 MHz, CDCl_3_) δ: 34.33 (q, *J* = 2 Hz, C3), 40.11 (C2), 95.47 (q, *J* = 26 Hz, C16), 104.79 (C8), 112.71 (C12), 114.01 (C6), 117.52 (C4), 118.82 (C14), 121.98 (q, *J* = 229 Hz, C17), 122.49 (C13), 127.41 (C7), 129.84 (C15), 130.75 (C10), 150.13 (C5), 152.30 (C11), 156.88 (C9), 168.02 (C1); IR (KBr) ν_max_ (cm^−1^): 3405 (OH), 1723 (C=O), 1625, 1597 (Ar), 758 (CF_3_); HRMS (ESI): *m*/*z* calcd for C_17_H_10_F_3_O_5_ [M − H]^+^: 351.0480; found: 351.0236.

*2,7-Dihydroxy-9-methoxy-7-(trifluoromethyl)-6a,12b-dihydro-6H,7H-chromeno[3,4-c]chromen-6-one* (**3n**): Eluent: DCM:PE:AcOH = 4:8:1; yellowish crystal; M.p. 187.8~188.5 °C; ^1^H-NMR (DMSO-*d_6_*, 400 MHz) δ: 3.51 (d, *J* = 4 Hz, 1H, CH), 3.79 (s, 3H, CH_3_), 4.82 (d, *J* = 4 Hz, 1H, CH), 6.23 (s, 1H, -OH), 6.60 (dd, *J*_1_ = 8 Hz, *J*_2_ = 1.6 Hz, 1H, Ar-H), 6.87 (d, *J* = 8 Hz, 1H, Ar-H), 6.98~7.16 (m, 4H, Ar-H), 8.62 (s, 1H, Ar-H), 9.24 (s, 1H, -OH); ^13^C NMR (100 MHz, DMSO-d^6^) δ: 33.73 (C3), 42.18 (C2), 56.21 (-OCH_3_), 95.23 (q, *J* = 26 Hz, C16), 112.63 (C13), 112.98 (C7), 114.68 (C9), 116.71 (C15), 118.36 (C14), 121.27 (C6), 122.65 (C10), 122.69 (q, *J* = 289 Hz, C17), 124.47 (C4), 140.17 (C5), 144.60 (C12), 148.90 (C11), 154.33 (C8), 164.44 (C1); IR (KBr) ν_max_ (cm^−1^): 3453 (OH), 1728 (C=O), 1605 (Ar), 730 (CF_3_); HRMS (ESI): *m*/*z* calcd for C_18_H_12_F_3_O_6_ [M − H]^+^: 381.0586; found: 381.0317.

*11-Chloro-2,7-dihydroxy-7-(trifluoromethyl)-6a,12b-dihydro-6H,7H-chromeno[3,4-c]chromen-6-one* (**3o**): Eluent: DCM:PE:AcOH = 4:8.5:0.5; white solid; M.p. 192.5~193.3 °C; ^1^H-NMR (DMSO-*d*_6_, 400 MHz) δ: 3.61 (d, *J* = 4 Hz, 1H, CH), 4.86 (d, *J* = 4 Hz, 1H, CH), 6.25 (s, 1H, -OH), 6.62 (d, *J* = 8 Hz, 1H, Ar-H), 6.90 (d, *J* = 8 Hz, 1H, Ar-H), 7.07 (d, *J* = 12 Hz, 1H, Ar-H), 7.43 (dd, *J*_1_ = 8 Hz, *J*_2_ = 2.4 Hz, 1H, Ar-H), 7.59 (s, 1H, Ar-H), 9.32 (s, 1H, -OH); ^13^C-NMR (100 MHz, DMSO-*d*_6_) δ: 33.46 (C3), 41.48 (C2), 95.35 (q, *J* = 32 Hz, C16), 112.77 (C12), 114.82 (C7), 116.88 (C9), 119.54 (C6), 122.25 (C14), 122.49 (q, *J* = 288 Hz, C17), 123.93 (C13), 126.56 (C15), 129.84 (C10), 130.79 (C4), 144.51 (C5), 149.64 (C11), 154.41 (C8), 164.13 (C1); IR (KBr) ν_max_ (cm^−1^): 3273 (OH), 1731 (C=O), 1611, 1482 (Ar), 825 (CF_3_); HRMS (ESI): *m/z* calcd for C_17_H_9_ClF_3_O_5_ [M − H]^+^: 385.0091; found: 384.9819.

*1,7-Dihydroxy-3-methyl-7-(trifluoromethyl)-6a,12b-dihydro-6H,7H-chromeno[3,4-c]chromen-6-one* (**3p**)**:** Crystallization from ethyl acetate-petroleum ether, white solid; M.p. 170.8~171.5 °C; 1H-NMR (CDCl_3_, 400 MHz) δ: 2.42 (s, 3H, CH_3_), 3.53 (d, *J* = 4 Hz, 1H, CH), 4.58 (d, *J* = 4 Hz, 1H, CH), 5.02 (s, 1H, -OH), 6.46 (s, 1H, -OH), 6.59 (dt, *J*_1_ = 8 Hz, *J*_2_ = 0.8 Hz, 1H, Ar-H), 6.67 (d, *J* = 2.4 Hz, 1H, Ar-H), 6.86 (td, *J*_1_ = 4 Hz, *J*_2_ = 0.8 Hz, 1H, Ar-H), 7.03 (dd, *J*_1_ = 8 Hz, *J*_2_ = 0.8 Hz, 1H, Ar-H), 7.21 (d, *J* = 4 Hz, 1H, Ar-H), 7.43 (d, *J* = 0.8 Hz, 1H, Ar-H); ^13^C-NMR (100 MHz, CDCl_3_) δ: 18.95 (-CH_3_), 31.20 (C3), 39.83 (C2), 95.82 (q, *J* = 26 Hz, C16), 102.53 (C12), 113.25 (C8), 114.46 (C6), 117.66 (C4), 118.43 (C14), 122.02 (q, *J* = 229 Hz, C17), 122.66 (C13), 126.75 (C15), 129.86 (C10), 138.93 (C7), 150.35 (C5), 152.57 (C11), 156.21 (C9), 168.39 (C1); IR (KBr) _νmax_ (cm^−1^): 3461 (OH), 1706 (C=O), 1633, 1594 (Ar), 753 (CF_3_); HRMS (ESI): *m*/*z* calcd for C_18_H_12_F_3_O_5_ [M − H]^+^: 365.0637; found: 365.0378.

## 4. Conclusions

In conclusion, we have developed a novel strategy to obtain chromeno[3,4-c]chromen-6-ones **3a**–**p**, a new kind of dihydrocoumarins, from the reactions of 3-trifluoroacetylcoumarin with phenols in one step. The chemical structures of the title compounds have been verified with the aid of several spectroscopic methods. The X-ray crystal structures of compounds **3a** and **3n** showed the twisted conformation in their cyclic structures. The antifungal activity of the compounds **3a**–**p** was assessed against two fungal strains with the mycelial growth rate method. The preliminary results indicated that the phenyl ring at the dihydrocoumarins favors activity against *Fusarium monitiforme.* Compound **3l** displayed the highest antifungal activity of 84.6% against *Fusarium monitiforme*. We continue our efforts to obtain new potent, broad spectrum and safe fluorine-bearing coumarins as antifungal drug-like candidates.

## Figures and Tables

**Table 1:** The screening of catalysts and acidifiers ^a^.

Entry	Base	Acid	t (h)	Yield ^b^ (%)
1	i-Pr_2_NEt	HCl	2	85
2	NEt_3_	HCl	2	85
3	DBU	HCl	2	80
4	DMAP	HCl	2	4
5	NMI	HCl	2	3
6	Py	HCl	2	trace
7	NMM	HCl	2	62
8	TMEDA	HCl	2	29
9	DABCO	HCl	2	13
10	K_2_CO_3_	HCl	2	trace
11	K_2_CO_3_ + TEBA	HCl	2	24
12	KF	HCl	2	8
13	NEt_3_	Conc. H_2_SO_4_	2	85
14	NEt_3_	TFA	2	74
15	NEt_3_	*p*-TsOH	2	20
16	NEt_3_	Citric acid	2	38
17	NEt_3_	Conc. H_2_SO_4_	DCE	78
18	NEt_3_	Conc. H_2_SO_4_	THF	24
19	NEt_3_	Conc. H_2_SO_4_	CHCl_3_	7
20	NEt_3_	Conc. H_2_SO_4_	dioxane	3
21 ^c^	NEt_3_	Conc. H_2_SO_4_	CH_3_CN	85
22 ^d^	NEt_3_	Conc. H_2_SO_4_	CH_3_CN	65
23 ^e^	NEt_3_	Conc. H_2_SO_4_	CH_3_CN	71
24 ^c,f^	NEt_3_	Conc. H_2_SO_4_	CH_3_CN	86
25 ^c,g^	NEt_3_	Conc. H_2_SO_4_	CH_3_CN	87
26 ^c,h^	NEt_3_	Conc. H_2_SO_4_	CH_3_CN	87
27 ^c,i^	NEt_3_	Conc. H_2_SO_4_	CH_3_CN	89
28 ^c,j^	NEt_3_	Conc. H_2_SO_4_	CH_3_CN	93
29 ^c,k^	NEt_3_	Conc. H_2_SO_4_	CH_3_CN	92

^a^ Reaction conditions: **1a** (0.50 mmol), **2a** (0.50 mmol), catalyst (0.50 mmol), acidifier (0.60 mmol) and dichloromethane (5 mL), 45 °C, 2 h, sealed tube. ^b^ Isolated yields. ^c, d, e^ Run at 45 ºC, 25 °C, 65 °C ^f^ n(**2a**):n(**1a**):n(NEt_3_) = 1:1.1:1. ^g^ n(**2a**):n(**1a**):n(NEt_3_) = 1:1.2:1. ^h^ n(**2a**):n(**1a**):n(NEt_3_) = 1:1.3:1. ^i^ n(**2a**):n(**1a**):n(NEt_3_) = 1:1.2:1.3. ^j^ n(**2a**):n(**1a**):n(NEt_3_) = 1:1.2:1.4. ^k^ n(**2a**):n(**1a**):n(NEt_3_) = 1:1.2:1.5.

**Table 2:** Synthesis of compounds **3a**–**p**.

Entry	R_1_	R_2_	R_3_	T (°C)	t/h	Product ^a^	Yield ^b^ (%)
1	H	H	-	45	1.5	**3a**	93
2	8-OCH_3_	H	-	45	0.3	**3b**	90
3	7-OCH_3_	H	-	45	1.5	**3c**	94
4	6-CH_3_	H	-	45	1.5	**3d**	91
5	6-Br	H	-	45	0.5	**3e**	92
6	H	Br	-	45	1.5	**3f**	94
7	8-OCH_3_	Br	-	45	1	**3g**	89
8	7-OCH_3_	Br	-	45	1	**3h**	90
9	6-Cl	Br	-	45	1	**3i**	91
10	H	CN	-	45	2	**3j**	88
11	7-OCH_3_	CN	-	45	2	**3k**	89
12 ^c^	H	-	H	65	5	**3l**	65
13 ^c^	H	-	3-OH	65	3	**3m**	74
14 ^c^	8-OCH_3_	-	4-OH	65	6	**3n**	80
15 ^c^	6-Cl	-	4-OH	65	4	**3o**	82
16 ^c^	H	-	3-OH, 5-CH_3_	65	6	**3p**	87

^a^ Reaction conditions: **1a** (0.64 mmol), **2a** (0.50 mmol) and NEt_3_ (0.71 mmol) carried out in a pressure vial at 45 °C by high-performance liquid chromatography (HPLC) tracking. ^b^ Yield of isolated product. ^c^ Run at 65 °C.

## Supplementary Materials

The following are available online: Figure S1: Molecular packing of compound **3a** (CCDC 1900313) and **3n** (CCDC1900314); Table S1: The refinement information and crystallographic data of **3a** and **3n**; and Figures S2–S17:^1^H-NMR, ^13^C-NMR, and HRMS spectra of compounds **3a**–**p**.

## References

[1] Ayati A., Bakhshaiesh T.O., Moghimi S., Esmaeili R., Majidzadeh-A K., Safavi M., Firoozpour L., Emami S., Foroumadi A. (2018). Synthesis and biological evaluation of new coumarins bearing 2,4-diaminothiazole-5-carbonyl moiety. Eur. J. Med. Chem..

[2] Chauhan N.B., Patel N.B., Patel V.M., Mistry B.M. (2018). Synthesis and biological evaluation of coumarin clubbed thiazines scaffolds as antimicrobial and antioxidant. Med. Chem. Res..

[3] Al-Amiery A.A., Al-Majedy Y.K., Kadhum A.A., Mohamad A.B. (2015). Novel macromolecules derived from coumarin: Synthesis and antioxidant activity. Sci. Rep..

[4] Ambekar S.P., Dhananjaya M.C., Arunkumar S., Kumar M.K., Shobith R., Surender M., Obelannavar K., Rangappa K.S. (2017). Synthesis of coumarin-benzotriazole hybrids and evaluation of their anti-tubercular activity. Lett. Org. Chem..

[5] Amin K.M., Taha A.M., George R.F., Mohamed N.M., Elsenduny F.F. (2018). Synthesis, antitumor activity evaluation, and DNA-binding study of coumarin-based agents. Arch. Pharm..

[6] Zhang R.R., Liu J., Zhang Y., Hou M.Q., Zhang M.Z., Zhou F.G., Zhang W.H. (2016). Microwave-assisted synthesis and antifungal activity of novel coumarin derivatives: Pyrano[3,2-c]chromene-2,5-diones. Eur. J. Med. Chem..

[7] Shi X., Lv C.W., Li J., Hou Z., Xiao Hui Y., Zhang Z.D., Luo X.X., Yuan Z., Li M.K. (2015). Synthesis, photoluminescent, antibacterial activities and theoretical studies of three novel coumarin and dihydropyran derivatives containing a triphenylamine group. Res. Chem. Intermed..

[8] Shen Y.F., Liu L., Feng C.Z., Yang H., Chen C., Wang G.X., Zhu B. (2018). Synthesis and antiviral activity of a new coumarin derivative against spring viraemia of carp virus. Fish Shellfish Immun..

[9] Sangshetti J.N., Khan F.A.K., Kulkarni A.A., Patil R.H., Pachpinde A.M., Lohar K.S., Shinde D.B. (2016). Antileishmanial activity of novel indolyl–coumarin hybrids: Design, synthesis, biological evaluation, molecular docking study and in silico ADME prediction. Bioorg. Med. Chem. Lett..

[10] Morsy S.A., Farahat A.A., Nasr M.N.A., Tantawy A.S. (2017). Synthesis, molecular modeling and anticancer activity of new coumarin containing compounds. Saudi. Pharm. J..

[11] Wu J., Peng T., Fang C., Leng Y., Tong L., Li M., Rong Q., Hua X., Jian D., Duan W. (2015). Synthesis and Anticancer Activity of 7,8-dihydroxy-4-arylcoumarins. Lett. Drug. Des. Discov..

[12] Sun J., Ding W.X., Hong X.P., Zhang K.Y. (2014). Antioxidant and antitumor activities of 4-arylcoumarins and 4-aryl-3,4-dihydrocoumarins. Biochimie.

[13] Sun J., Ding W.X., Hong X.P., Zhang K.Y. (2012). Synthesis and antimicrobial activities of 4-aryl-3,4-dihydrocoumarins and 4-arylcoumarins. Chem. Nat. Compd..

[14] Pierson J.T., Dumètre A., Hutter S., Delmas F., Laget M., Finet J.P., Azas N., Combes S. (2010). Synthesis and antiprotozoal activity of 4-arylcoumarins. J. Cheminf..

[15] Taechowisan T., Lu C.h., Shen Y.m., Lumyong A. (2005). Secondary metabolites from endophytic Streptomyces aureofaciens CMUAc130 and their antifungal activity. Microbiology.

[16] Jung J.W., Kim N.J., Yun H.Y., Han Y. (2018). Recent advances in synthesis of 4-arylcoumarins. Molecules.

[17] Pourshojaei Y., Jadidi M.H., Eskandari K., Foroumadi A., Asadipour A. (2018). An eco-friendly synthesis of 4-aryl-substituted pyrano-fuzed coumarins as potential pharmacological active heterocycles using molybdenum oxide nanoparticles as an effective and recyclable catalyst. Res. Chem. Intermed..

[18] El-Dean A.M.K., Zaki R.M., Geies A.A., Radwan S.M., Tolba M.S. (2013). Synthesis and antimicrobial activity of new heterocyclic compounds containing thieno[3,2-]coumarin and pyrazolo[4,3-]coumarin frameworks. Russ. J. Bioorg. Chem..

[19] Yin S., Yang X.Y., Zhang M., Zhou Z.Q., XU H.H. (2013). Synthesis and fungicidal activities of novel fluorine-containing pyrimithamine derivatives. Chin. J. Synth. Chem..

[20] Iaroshenko V.O., Ali S., Babar T.M., Dudkin S., Mkrtchyan S., Rama N.H., Villinger A., Langer P. (2011). 4-Chloro-3-(trifluoroacetyl)coumarin as a novel building block for the synthesis of 7-(trifluoromethyl)-6H-chromeno[4,3-b]quinolin-6-ones. Tetrahedron Lett..

[21] Vdovenko S.I., Gerus I.I., Kukhar V.P. (2007). Steric effects on the mechanism of reaction of nucleophilic substitution of β-substituted alkoxyvinyl trifluoromethyl ketones with four secondary amines. J. Phys. Org. Chem..

[22] Huang D.F., Zhang X.H., Wang K.H., Su Y.P., Hu Y.L. (2018). Synthesis of 2-trifluoromethyl-2,3-dihydro-1,3,4-oxadiazole derivatives. J. Northwest Normal Univ..

[23] Xu C.L., Yang G.Y., Zhao M.Q., Wang C.X., Fan S.F., Xie P.H., Li X. (2016). Microwave assisted one-pot synthesis of novel trifluoromethyl coumarin thiosemicarbazones and their antifungal activities. Curr. Microw. Chem..

[24] Yang G.Y., Yang J.T., Wang C.X., Fan S.F., Xie P.H., Xu C.L. (2014). Microwave-assisted TsOH/SiO_2_-catalyzed one-pot synthesis of novel fluoro-substituted coumarin hydrazones under solvent-free conditions. J. Fluor. Chem..

[25] Fan H.F., Wang X.W., Zhao J.W., Li X.J., Gao J.M., Zhu S.Z. (2013). Synthesis of 3-trifluoromethyl-substituted benzo[f]chromene derivatives in a one-pot reaction. Synth. Commun..

[26] Xian D.W. (2011). Biological Activities Study on 4- arylcoumarins and 4-aryl-3,4 dihydrocoumarins.

[27] Zhang J., Yang Y., Liu X.H., Zhu H.T. Progress in synthesis of chiral dihydrocoumarin derivatives. Proceedings of the 3rd Symposium on Molecular Chirality in China.

[28] Wallace K.J., Belcher W.J., Turner D.R., Syed K.F., Steed J.W. (2003). Slow anion exchange, conformational equilibria, and fluorescent sensing in venus flytrap aminopyridinium-based anion hosts. J. Am. Chem. Soc..

[29] Huang Z.X. (1993). Plant Chemical Protection Experiment Instruction.

[30] Yang G.Y., Wang C.X., Fan S.F., Xie P.H., Jin Q., Xu C.L. (2015). Microwave assisted solvent-free synthesis of 3-(trifluoroacetyl)coumarins. Chin. J. Org. Chem..

