# Supplementary material for: Novel 6a,12b-Dihydro-6H,7H-chromeno[3,4-c] chromen-6-ones: Synthesis, Structure and Antifungal Activity
Source: Molecules. 2019 May 5;24(9):1745. doi: 10.3390/molecules24091745 (PMC6539249; doi:10.3390/molecules24091745)

Supporting Information

Synthesis and antifungal activity of novel coumarin derivatives: chromeno[3,4-c]chromen-6-ones

Jin-ping Bao [1], Cui-lian Xu [2,*] and Guo-yu Yang [2,*]

1 **Content**……………………………………………………1

2 **Characterization Data**…………………………………2

3 **^{1}H and ^{13}C NMR Spectra**………………………………9

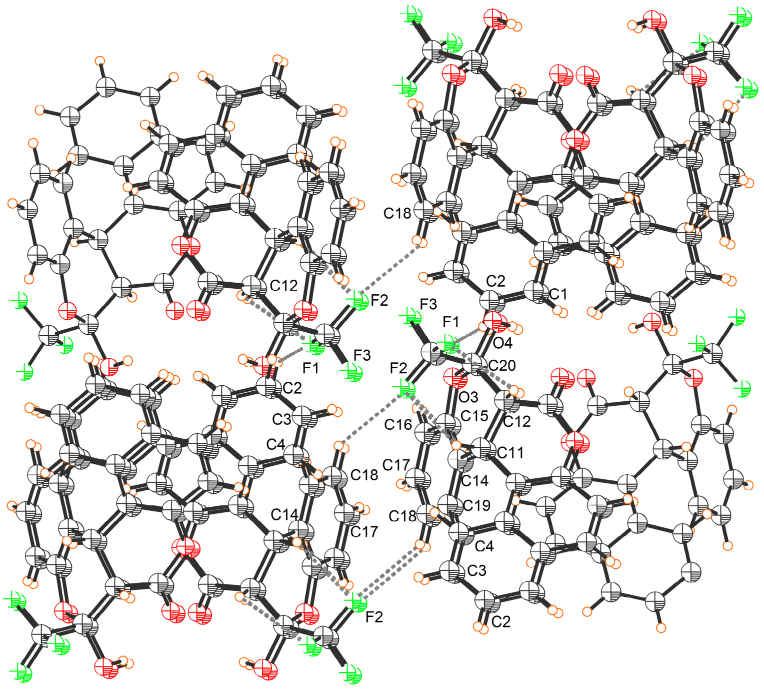

3a

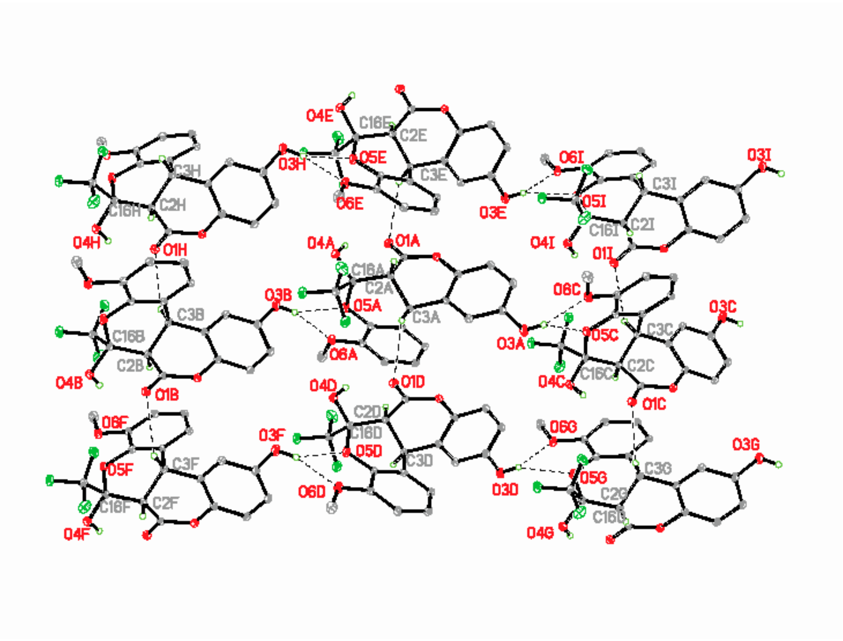

3n

Figure S1: Molecular packing of compound **3a** (CCDC 1900313) and **3n**(CCDC1900314) along b axis

Table S1 The refinement information and crystallographic data of 3a and 3n

Empirical formula	$C_{21}H_{13}F_3O_4$ **(3a)**	$C_{18}H_{13}F_3O_6$ **(3n)**
Formula weight	386.31	382.28
Temperature/K	293(2)	293(2)
Crystal system	orthorhombic	orthorhombic
Space group	Pbca	Pbca
a/Å	8.6244(2)	15.2410(6)
b/Å	17.4245(4)	11.6170(4)
c/Å	22.5188(6)	19.1620(6)
α/°	90	90
β/°	90	90
γ/°	90	90
Volume/Å3	3384.02(14)	3392.7(2)
Z	8	8
ρcalcg/cm3	1.517	1.497
μ/mm-1	1.089	1.166
F(000)	1584.0	1568.0
Crystal size/mm3	0.23 × 0.2 × 0.18	0.16 × 0.15 × 0.13
Radiation	CuKα (λ = 1.54184)	CuKα (λ = 1.54184)
2Θ range for data collection/°	7.852 to 134.136	9.23 to 134.142
Index ranges	-7 ≤ h ≤ 10, -20 ≤ k ≤ 13, -21 ≤ l ≤ 26	-16 ≤ h ≤ 18, -13 ≤ k ≤ 12, -14 ≤ l ≤ 22
Reflections collected	7623	7504

Independent reflections	3023 [Rint = 0.0236, Rsigma = 0.0307]	3029 [Rint = 0.0254, Rsigma = 0.0298]
Data/restraints/parameters	3023/0/257	3029/0/248
Goodness-of-fit on F2	1.041	1.034
Final R indexes [I>=2σ (I)]	R1 = 0.0469, wR2 = 0.1174	R1 = 0.0437, wR2 = 0.1157
Final R indexes [all data]	R1 = 0.0603, wR2 = 0.1290	R1 = 0.0551, wR2 = 0.1255
Largest diff. peak/hole / e Å-3	0.15/-0.26	0.23/-0.18

***2-Hydroxy-2-(trifluoromethyl)-2a,10c-dihydro-2H,3H-benzo[f]chromeno[3,4-c]chromen-3-one*(3a):**

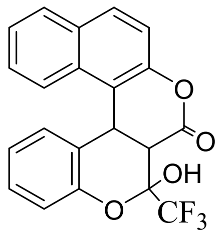

^{1}H-NMR ($CDCl_3$, 400 MHz) δ: 3.72 (d, J = 4 Hz, 1H, CH), 5.26 (d, J = 4 Hz, 1H, CH), 6.59 (d, J = 4 Hz, 1H, Ar-H), 6.70 (td, J_1 = 8 Hz, J_2 = 1.2 Hz, Ar-H), 7.12 (q, J = 4 Hz, 1H, Ar-H), 7.23~7.33 (m, 2H, Ar-H), 7.48 (s, 1H, -OH), 7.64 (td, J_1 = 8 Hz, J_2 = 0.8 Hz, 1H, Ar-H), 7.74 (td, J_1 = 8 Hz, J_2 = 1.2 Hz, 2H, Ar-H), 7.98~8.06 (m, 3H, Ar-H); ^{13}C-NMR (100 MHz, $CDCl_3$) δ: 31.12 (q, J = 3 Hz, C1), 39.46 (C1), 95.71 (q, J = 33 Hz, C20), 116.24 (C16), 116.94 (C18), 117.60 (C8), 118.29 (C10), 122.13 (q, J = 286 Hz, C21), 122.64 (C4), 122.90 (C2), 126.20 (C3), 127.61 (C17), 128.61 (C7), 129.13 (C1), 129.92 (C19), 130.88 (C6), 131.33 (C14), 131.82 (C5), 147.44 (C9), 152.64 (C15), 168.35 (C13); IR (KBr) ν_{max} (cm^{-1}): 3419 (OH), 1728 (C=O), 1585 (Ar), 822 (CF_3); HRMS (ESI): *m/z* calcd for $C_{21}H_{12}F_3O_4$ $[M-H]^+$: 385.0688; found: 385.0688.

***2-Hydroxy-14-methoxy-2-(trifluoromethyl)-2a,10c-dihydro-2H,3H-benzo[f]chromeno[3,4-c]chromen-3-one* (3b):**

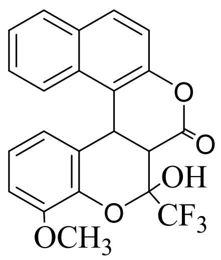

^{1}H-NMR ($CDCl_3$, 400 MHz) δ: 3.67 (d, J = 4 Hz, 1H, CH), 3.88 (s, 3H, CH_3), 5.22 (d, J = 4 Hz, 1H, CH), 6.13 (d, J = 4 Hz, 1H, Ar-H), 6.68 (t, J = 8 Hz, 1H, Ar-H), 6.81 (d, J = 8 Hz, 1H, Ar-H), 7.27 (d, J = 4 Hz, 1H, Ar-H), 7.47 (s, 1H, -OH), 7.58 (td, J_1 = 8 Hz, J_2 =0.8 Hz, 1H, Ar-H), 7.68 (td, J_1 = 8 Hz, J_2 = 0.8 Hz, 1H, Ar-H), 7.92~8.00 (m, 3H, Ar-H); ^{13}C-NMR (100 MHz, $CDCl_3$) δ: 31.16 (q, J = 2 Hz, C11), 39.38 (C12), 56.30 (-OCH_3), 95.97 (q, J = 26 Hz, C20), 112.34 (C17), 116.29 (C19), 116.87 (C18), 118.92 (C8), 119.40 (C10), 122.08 (q, J = 229 Hz, C21), 122.20 (C4), 122.90 (C2), 126.10 (C3), 128.49 (C7), 129.04 (C1), 130.79 (C6),, 131.27 (C14), 131.73 (C5), 142.31 (C16), 147.41 (C15), 148.65 (C9), 168.26 (C13); IR (KBr) ν_{max} (cm^{-1}): 3391 (OH), 1736 (C=O), 1582, 1478 (Ar), 817 (CF_3); HRMS (ESI): *m/z* calcd for $C_{22}H_{15}F_3O_5$ $[M-H]^+$: 415.0793; found: 415.0795.

2-Hydroxy-13-methoxy-2-(trifluoromethyl)-2a,10c-dihydro-2H,3H-benzo[f]chromeno[3,4-c]chromen-3-one **(3c):**

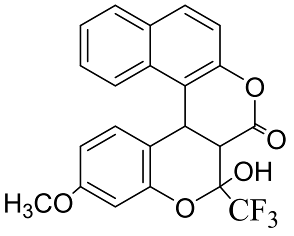

^{1}H-NMR ($CDCl_3$, 400 MHz) δ: 3.64 (d, J = 4 Hz, 1H, CH), 3.72 (s, 3H, CH_3), 5.14 (d, J = 4 Hz, 1H, CH), 6.31 (dd, J_1 = 8 Hz, J_2 = 2 Hz, 1H, Ar-H), 6.42 (dd, J_1 = 4 Hz, J_2 = 0.8 Hz, 1H, Ar-H), 6.62 (d, J = 4 Hz, 1H, Ar-H), 7.69 (td, J_1 = 8 Hz, J_2 = 0.8 Hz, 1H, Ar-H), 7.92~8.00 (m, 3H, Ar-H); ^{13}C-NMR (100 MHz, $CDCl_3$) δ: 30.59 (q, J = 3 Hz, C11), 39.64 (C12), 55.39 (-OCH_3), 95.79 (q, J = 26 Hz, C20), 102.11 (C16), 109.75 (C18), 110.00 (C8), 116.38 (C14), 116.90 (C10), 122.05 (q, J = 229 Hz, C21), 122.87 (C4), 126.11 (C2), 128.25 (C3), 128.51 (C7), 129.06 (C1), 130.64 (C19), 131.29 (C6), 131.76 (C5), 147.28 (C9), 153.55 (C15), 160.87 (C17), 168.41 (C13); IR (KBr) ν_{max} (cm^{-1}): 3402 (OH), 1736 (C=O), 1628, 1501 (Ar), 819 (CF_3); HRMS (ESI): *m/z* calcd for $C_{22}H_{15}F_3O_5$ $[M-H]^+$: 415.0793; found: 415.0792.

2-Hydroxy-12-methyl-2-(trifluoromethyl)-2a,10c-dihydro-2H,3H-benzo[f]chromeno[3,4-c]chromen-3-one (**3d**):

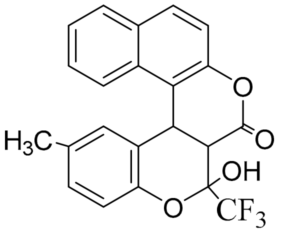

^{1}H-NMR (DMSO-d_6, 400 MHz) δ: 1.97 (s, 3H, CH_3), 3.61 (dd, J_1 = 6 Hz, J_2 = 1.2 Hz, 1H, CH), 5.60 (d, J = 6 Hz, 1H, CH), 6.17 (s, 1H, Ar-H), 7.02 (q, J = 8 Hz, 2H, Ar-H, OH), 7.25 (d, J = 8 Hz, 1H, Ar-H), 7.52 (t, J = 8 Hz, 1H, Ar-H), 7.62 (t, J = 8 Hz, 1H, Ar-H), 7.92 (d, J = 8 Hz, 1H, Ar-H), 8.00 (q, J = 4 Hz, 2H, Ar-H), 8.73 (d, J = 4 Hz, 1H, Ar-H); ^{13}C-NMR (100 MHz, DMSO-d_6) δ: 20.89 (-CH_3), 29.71 (C11), 41.54 (C12), 94.85 (q, J = 32 Hz, C20), 112.86 (C16), 115.82 (C8), 118.63 (C10), 122.66 (q, J = 288 Hz, C21), 123.31 (C4), 123.43 (C2), 125.11 (C3), 126.95 (C17), 128.00 (C7), 128.85 (C1), 129.10 (C18), 129.83 (C19), 130.61 (C14), 132.80 (C6), 133.52 (C5), 148.59 (C9), 150.30 (C15) (C13), 164.50 (C=O); IR (KBr) ν_{max} (cm^{-1}): 3287 (OH), 1765 (C=O), 1628, 1600 (Ar), 812 (CF_3); HRMS (ESI): *m/z* calcd for $C_{22}H_{15}F_3O_4$ $[M-H]^+$: 399.0844; found: 399.0805.

***12-Bromo-2-hydroxy-2-(trifluoromethyl)-2a,10c-dihydro-2H,3H-benzo[f]chromeno[3,4-c]chromen-3-one* (3e):**

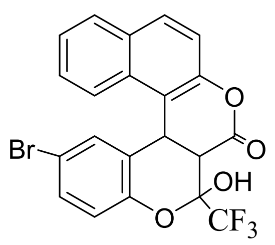

^{1}H-NMR ($CDCl_3$, 400 MHz) δ: 3.65 (d, J = 4 Hz, 1H, CH), 5.18 (d, J = 4 Hz, 1H, CH), 7.64 (d, J = 4 Hz, 1H, Ar-H), 6.97 (d, J = 4 Hz, 1H, Ar-H), 7.28~7.32 (m, 2H, Ar-H), 7.42 (s, 1H, -OH), 7.61 (t, J = 4 Hz, 1H, Ar-H), 7.72 (t, J = 4 Hz, 1H, Ar-H), 7.95~7.99 (m, 3H, Ar-H); ^{13}C NMR (100 MHz, $CDCl_3$) δ: 30.96 (C11), 39.14 (C12), 95.84 (q, J = 26 Hz, C20), 115.03 (C16), 115.32 (C18), 116.88 (C8), 119.44 (C10), 120.78 (C4), 121.93 (q, J = 229 Hz, C21), 122.47 (C2), 126.36 (C3), 128.90 (C7), 129.26 (C1), 130.11 (C17), 131.28 (C6), 131.39 (C5), 131.49 (C14), 132.99 (C19), 147.43 (C9), 151.76 (C15), 168.00 (C13); IR (KBr) ν_{max} (cm^{-1}): 3408 (OH), 1740 (C=O), 1628, 1471 (Ar), 809 (CF_3); HRMS (ESI): *m/z* calcd for $C_{21}H_{11}BrF_3O_4$ $[M-H]^+$: 462.9793; found: 462.9793.

***7-Bromo-2-hydroxy-2-(trifluoromethyl)-2a,10c-dihydro-2H,3H-benzo[f]chromeno[3,4-c]chromen-3-one* (3f):**

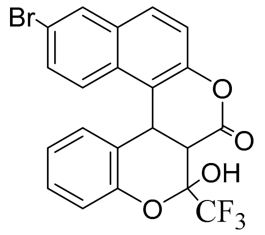

^{1}H-NMR ($CDCl_3$, 400 MHz) δ: 3.12 (d, J = 6 Hz, 1H, CH), 5.19 (d, J = 6 Hz, 1H, CH), 6.53 (d, J = 8 Hz, 1H, Ar-H), 6.80 (t, J = 8 Hz, 1H, Ar-H), 7.11 (d, J = 8 Hz, 1H, Ar-H), 7.26 (t, J = 8 Hz, 1H, Ar-H), 7.34 (d, J = 2 Hz, 1H, Ar-H), 7.41 (s, 1H, -OH), 7.80 (d, J = 8 Hz, 1H, Ar-H), 7.88~7.92 (t, J = 8 Hz, 2H, Ar-H), 8.17 (s, 1H, Ar-H); ^{13}C NMR (100 MHz, $CDCl_3$) δ: 31.19 (d, J = 3 Hz, C11), 39.37 (C12), 95.65 (q, J = 33 Hz, C20), 116.57 (C16), 117.74 (C18), 117.93 (C1), 118.21 (C8), 120.26 (C10), 122.09 (q, J = 287 Hz, C21), 122.73 (C4), 124.67 (C3), 127.37 (C7), 129.93 (C17), 130.10 (C19), 130.41 (C6), 131.11 (C2), 131.94 (C14), 132.39 (C5), 147.58 (C9), 152.61 (C15), 167.96 (C13); IR (KBr) ν_{max} (cm^{-1}): 3360 (OH), 1723 (C=O), 1583, 1479 (Ar), 758 (CF_3); HRMS (ESI): *m/z* calcd for $C_{21}H_{11}BrF_3O_4$ $[M-H]^+$: 462.9793; found: 462.9791.

***7-Bromo-2-hydroxy-14-methoxy-2-(trifluoromethyl)-2a,10c-dihydro-2H,3H-benzo[f]chromeno[3,4-c]chromen-3-one* (3g):**

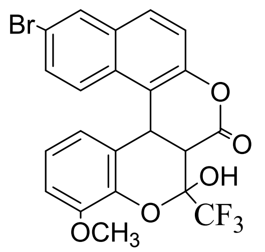

^{1}H-NMR ($CDCl_3$, 400 MHz) δ: 3.72 (d, J = 4 Hz, 1H, CH), 5.19 (d, J = 4 Hz, 1H, CH), 6.09 (d, J = 8 Hz, 1H, Ar-H), 7.33 (d, J = 8 Hz, 1H, Ar-H), 7.45 (s, 1H, -OH), 7.79 (d, J = 8 Hz, 1H, Ar-H), 7.89 (t, J = 8 Hz, 2H, Ar-H), 8.16 (s, 1H, Ar-H); ^{13}C-NMR (100 MHz, $CDCl_3$) δ: 31.28 (q, J = 3 Hz, C11), 39.33 (C12), 56.35 (-OCH_3), 95.95 (q, J = 33 Hz, C20), 112.51 (C17), 116.67 (C19), 118.18 (C18), 118.69 (C1), 119.07 (C8), 120.20 (C10), 122.07 (q, J = 286 Hz, C21), 122.34 (C4), 124.72 (C3), 129.88 (C7), 130.37 (C6), 131.05 (C2), 131.87 (C14), 132.37 (C5), 142.33 (C16), 147.62 (C15), 148.77 (C9), 167.90 (C13); IR (KBr) ν_{max} (cm^{-1}): 3416 (OH), 1762 (C=O), 1580 (Ar), 865 (CF_3); HRMS (ESI): *m/z* calcd for $C_{22}H_{14}BrF_3O_5$ $[M - H]^+$: 492.9898; found: 492.9850.

***7-Bromo-2-hydroxy-13-methoxy-2-(trifluoromethyl)-2a,10c-dihydro-2H,3H-benzo[f]chromeno[3,4-c]chromen-3-one* (3h):**

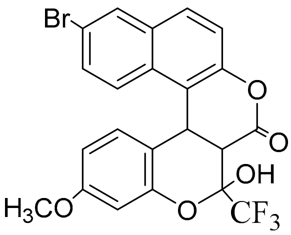

^{1}H-NMR ($CDCl_3$, 400 MHz) δ: 3.65 (d, J = 8 Hz, 1H, CH), 3.74 (s, 3H, CH_3), 5.08 (d, J = 8 Hz, 1H, CH), 6.32~6.38 (m, 2H, Ar-H), 6.63 (d, J = 4 Hz, 1H, Ar-H), 7.30 (d, J = 8 Hz, 1H, Ar-H), 7.37 (s, 1H, -OH), 7.76 (dd, J_1 = 8 Hz, J_2 = 2.4 Hz, 1H, Ar-H), 7.86 (t, J = 8 Hz, 1H, Ar-H), 8.13 (d, J = 1.6 Hz, 1H, Ar-H); ^{13}C NMR (100 MHz, $CDCl_3$) δ: 30.68 (q, J = 3 Hz, C11), 39.57 (C12), 55.45 (-OCH_3), 95.42 (q, J = 33 Hz, C20), 102.23 (C16), 109.62 (C18), 109.89 (C1), 116.74 (C8), 118.19 (C14), 120.20 (C10), 122.03 (q, J = 286 Hz, C21), 124.67 (C4), 128.08 (C3), 129.79 (C7), 130.37 (C6), 131.07 (C2), 131.88 (C19), 132.37 (C5), 147.48 (C9), 153.57 (C15), 161.02 (C17), 168.08 (C13); IR (KBr) ν_{max} (cm^{-1}): 3419 (OH), 1737 (C=O), 1580, 1499 (Ar), 812 (CF_3); HRMS (ESI): *m/z* calcd for $C_{22}H_{14}BrF_3O_5$ $[M - H]^+$: 492.9898; found: 492.9510.

7-Bromo-12-chloro-2-hydroxy-2-(trifluoromethyl)-2a,10c-dihydro-2H,3H-benzo[f]chromeno[3,4-c]chromen-3-one **(3i)**:

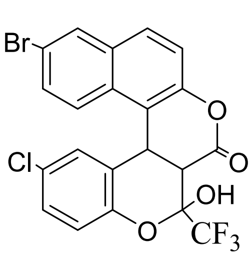

^{1}H-NMR ($CDCl_3$, 400 MHz) δ: 3.70 (d, J = 6 Hz, 1H, CH), 5.15 (d, J = 6 Hz, 1H, CH), 6.47 (s, 1H, Ar-H), 7.06 (d, J = 8 Hz, 1H, Ar-H), 7.22 (d, J = 4 Hz, 1H, Ar-H), 7.35 (d, J = 8 Hz, 1H, Ar-H), 7.40 (s, 1H, -OH), 7.80~7.93 (m, 3H, Ar-H), 8.18 (s, 1H, Ar-H); ^{13}C-NMR (100 MHz, $CDCl_3$) δ: 31.09 (d, J = 2 Hz, C11), 39.11 (C12), 95.83 (q, J = 33 Hz, C20), 115.72 (C16), 118.20 (C1), 119.21 (C8), 119.69 (C10), 120.48 (C4), 121.94 (q, J = 286 Hz, C21), 124.26 (C3), 127.07 (C18), 127.86 (C7), 130.12 (C17), 130.27 (C19), 130.36 (C6), 131.30 (C2), 132.26 (C14), 132.47 (C5), 147.63 (C9), 151.22 (C15), 167.64 (C13); IR (KBr) ν_{max} (cm^{-1}): 3430 (OH), 1748 (C=O), 1583, 1504 (Ar), 812 (CF_3); HRMS (ESI): *m/z* calcd for $C_{21}H_{11}BrClF_3O_4$ $[M + H]^+$: 498.9560; found: 498.9331.

***2-Hydroxy-3-oxo-2-(trifluoromethyl)-2a,10c-dihydro-2H,3H-benzo[f]chromeno[3,4-c]chromene-8-carbonitrile* (3j):**

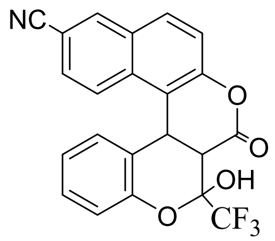

^{1}H-NMR ($CDCl_3$, 400 MHz) δ: 3.32 (dd, J_1 = 16 Hz, J_2 = 2 Hz, 1H, CH), 5.34 (d, J = 8 Hz, 1H, CH), 5.70 (s, 1H, -OH), 6.55 (dd, J_1 = 8 Hz, J_2 = 0.8 Hz, 1H, Ar-H), 6.70 (t, J = 8 Hz, 1H, Ar-H), 6.80 (d, J = 4 Hz, 1H, Ar-H), 7.09 (td, J_1 = 8 Hz, J_2 = 1.6 Hz, 1H, Ar-H), 7.48 (d, J = 8 Hz, 1H, Ar-H), 7.59 (dd, J_1 = 8 Hz, J_2 = 1.6 Hz, 1H, Ar-H), 7.87 (d, J = 12 Hz, 1H, Ar-H), 7.94 (d, J = 12 Hz, 1H, Ar-H), 8.24 (d, J = 0.8 Hz, 1H, Ar-H); ^{13}C-NMR (100 MHz, $CDCl_3$) δ: 31.41 (C11), 35.04 (C12), 95.53 (q, J = 33 Hz, C20), 108.76 (C1), 115.60 (C16), 118.15 (-CN), 118.87 (C18), 119.51 (C7), 122.42 (q, J = 286 Hz, C21), 121.47 (C8), 124.67 (C10), 125.98 (C3), 127.84 (C17), 128.04 (C19), 129.11 (C4), 130.00 (C5), 130.35 (C6), 132.86 (C14)), 134.54 (C2), 152.40 (C15, C9), 167.22 (C13); IR (KBr) ν_{max} (cm^{-1}): 3419 (OH), 2228 (CN), 1748 (C=O), 1628, 1585 (Ar), 753 (CF_3); HRMS (ESI): *m/z* calcd for $C_{22}H_{12}F_3NO_4$ $[M-H]^+$: 410.0640; found: 410.0600.

***2-Hydroxy-13-methoxy-3-oxo-2-(trifluoromethyl)-2a,10c-dihydro-2H,3H-benzo[f]chromeno[3,4-c]chromene-8-carbonitrile* (3k)**:

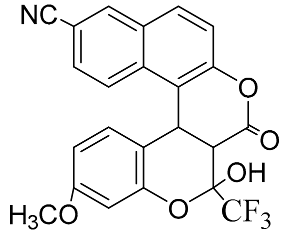

^{1}H-NMR ($CDCl_3$, 400 MHz) δ: 3.68 (d, J = 6 Hz, 1H, CH), 3.74 (s, 3H, CH_3), 5.13 (d, J = 6 Hz, 1H, CH), 5.30 (s, 1H, -OH), 6.34 (d, J = 4 Hz, 1H, Ar-H), 6.64 (d, J = 4 Hz, 1H, Ar-H), 7.25 (d, J = 4 Hz, 1H, Ar-H), 7.43 (d, J = 12 Hz, 1H, Ar-H), 7.85 (dd, J_1 = 12 Hz, J_2 = 2 Hz, 1H, Ar-H), 8.03 (d, J = 8 Hz, 1H, Ar-H), 8.11 (d, J = 4 Hz, 1H, Ar-H), 8.36 (s, 1H, Ar-H); ^{13}C NMR (100 MHz, $CDCl_3$) δ: 30.69 (q, J = 3 Hz, C11), 39.48 (C12), 55.47 (-OCH_3), 95.63 (q, J = 30 Hz, C20), 102.34 (C16), 109.10 (C18), 109.93 (C1), 109.99 (-CN), 117.02 (C7), 118.43 (C14), 119.06 (C8), 121.96 (q, J = 286 Hz, C21), 124.39 (C10), 127.86 (C3), 129.08 (C4), 130.17 (C19), 131.36 (C5), 133.48 (C6), 134.92 (C2), 149.44 (C9), 153.56 (C15), 161.14 (C17), 167.55 (C13); IR (KBr) ν_{max} (cm^{-1}): 3430 (OH), 2228 (CN), 1742 (C=O), 1619, 1580 (Ar), 817 (CF_3); HRMS (ESI): *m/z* calcd for $C_{23}H_{13}F_3NO_4$ $[M-H]^+$: 440.0746; found: 440.0743.

***7-Hydroxy-7-(trifluoromethyl)-6a,12b-dihydro-6H,7H-chromeno[3,4-c]chromen-6-one* (3l):**

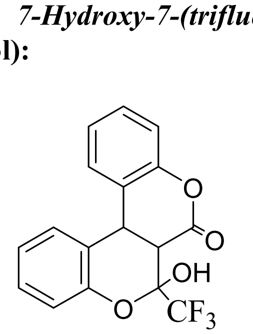

^{1}H-NMR ($CDCl_3$, 400 MHz) δ: 3.67 (d, J = 6 Hz, 1H, CH), 4.59 (d, J = 6 Hz, 1H, CH), 6.69 (d, J = 8 Hz, 1H, Ar-H), 6.91 (t, J = 8 Hz, 1H, Ar-H), 7.07 (d, J = 8 Hz, 1H, Ar-H), 7.16 (d, J = 8 Hz, 1H, Ar-H), 7.23~7.28 (m, 1H, Ar-H), 7.38 (t, J = 8 Hz, 1H, Ar-H), 7.46~7.52 (m, 2H, Ar-H); ^{13}C-NMR

(100 MHz, $CDCl_3$) δ: 34.98 (C3), 39.91 (C2), 95.50 (q, J = 32 Hz, C16), 117.47 (C12), 117.58 (C14), 118.41 (C6), 121.96 (C8), 122.01 (q, J = 287 Hz, C17), 122.54 (C13), 125.65 (C7), 127.45 (C15), 129.91 (C9), 129.99 (C10), 130.09 (C4), 149.42 (C5), 152.36 (C11), 168.11 (C1); IR (KBr) ν_{max} (cm^{-1}): 3414 (OH), 1726 (C=O), 1588, 1487 (Ar), 756 (CF_3); HRMS (ESI): *m/z* calcd for $C_{17}H_{10}F_3O_4$ $[M-H]^+$: 335.0531; found: 335.0298.

***1,7-Dihydroxy-7-(trifluoromethyl)-6a,12b-dihydro-6H,7H-chromeno[3,4-c]chromen-6-one*(3m)**

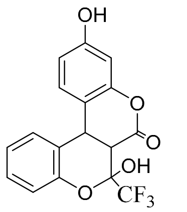

^{1}H-NMR ($CDCl_3$, 400 MHz) δ: 3.60 (d, J = 4 Hz, 1H, CH), 4.47 (d, J = 4 Hz, 1H, CH), 5.12 (s, 1H, -OH), 6.61 (d, J = 2 Hz, 1H, Ar-H), 6.75~6.80 (m, 2H, Ar-H), 6.87 (t, J = 4 Hz, 1H, Ar-H), 7.01 (d, J = 8 Hz, 1H, Ar-H), 7.20 (t, J = 4 Hz, 1H, Ar-H), 7.31 (d, J = 8 Hz, 1H, Ar-H); ^{13}C NMR (100 MHz, $CDCl_3$) δ: 34.33 (q, J = 2 Hz, C3), 40.11 (C2), 95.47 (q, J = 26 Hz, C16), 104.79 (C8), 112.71 (C12), 114.01 (C6), 117.52 (C4), 118.82 (C14), 121.98 (q, J = 229 Hz, C17), 122.49 (C13), 127.41 (C7), 129.84 (C15), 130.75 (C10), 150.13 (C5), 152.30 (C11), 156.88 (C9), 168.02 (C1); IR (KBr) ν_{max} (cm^{-1}): 3405 (OH), 1723 (C=O), 1625, 1597 (Ar), 758 (CF_3); HRMS (ESI): *m/z* calcd for $C_{17}H_{10}F_3O_5$ $[M-H]^+$: 351.0480; found: 351.0236.

***2,7-Dihydroxy-9-methoxy-7-(trifluoromethyl)-6a,12b-dihydro-6H,7H-chromeno[3,4-c]chromen-6-one* (3n):**

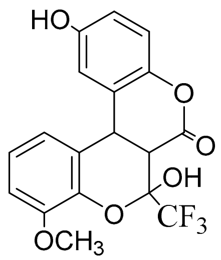

^{1}H-NMR (DMSO-d_6, 400 MHz) δ: 3.51 (d, J = 4 Hz, 1H, CH), 3.79 (s, 3H, CH_3), 4.82 (d, J = 4 Hz, 1H, CH), 6.23 (s, 1H, -OH), 6.60 (dd, J_1 = 8 Hz, J_2 = 1.6 Hz, 1H, Ar-H), 6.87 (d, J = 8 Hz, 1H, Ar-H), 6.98~7.16 (m, 4H, Ar-H), 8.62 (s, 1H, Ar-H), 9.24 (s, 1H, -OH); ^{13}C NMR (100 MHz, DMSO-d^6) δ: 33.73 (C3), 42.18 (C2), 56.21 (-OCH_3), 95.23 (q, J = 26 Hz, C16), 112.63 (C13), 112.98 (C7), 114.68 (C9), 116.71 (C15), 118.36 (C14), 121.27 (C6), 122.65 (C10), 122.69 (q, J = 289 Hz, C17), 124.47 (C4), 140.17 (C5), 144.60 (C12), 148.90 (C11), 154.33 (C8), 164.44 (C1); IR (KBr) ν_{max} (cm^{-1}): 3453 (OH), 1728 (C=O), 1605 (Ar), 730 (CF_3); HRMS (ESI): *m/z* calcd for $C_{18}H_{12}F_3O_6$ $[M-H]^+$: 381.0586; found: 381.0317.

***11-Chloro-2,7-dihydroxy-7-(trifluoromethyl)-6a,12b-dihydro-6H,7H-chromeno[3,4-c]chromen-6-one* (3o):**

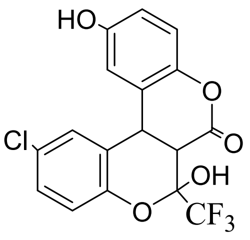

^{1}H-NMR (DMSO-d_6, 400 MHz) δ: 3.61 (d, J = 4 Hz, 1H, CH), 4.86 (d, J = 4 Hz, 1H, CH), 6.25 (s, 1H, -OH), 6.62 (d, J = 8 Hz, 1H, Ar-H), 6.90 (d, J = 8 Hz, 1H, Ar-H), 7.07 (d, J = 12 Hz, 1H, Ar-H), 7.43 (dd, J_1 = 8 Hz, J_2 = 2.4 Hz, 1H, Ar-H), 7.59 (s, 1H, Ar-H), 9.32 (s, 1H, -OH); ^{13}C-NMR (100 MHz, DMSO-d_6) δ: 33.46 (C3), 41.48 (C2), 95.35 (q, J = 32 Hz, C16), 112.77 (C12), 114.82 (C7), 116.88 (C9), 119.54 (C6), 122.25 (C14), 122.49 (q, J = 288 Hz, C17), 123.93 (C13), 126.56 (C15), 129.84 (C10), 130.79 (C4), 144.51 (C5), 149.64 (C11), 154.41 (C8), 164.13 (C1); IR (KBr) ν_{max} (cm^{-1}): 3273 (OH), 1731 (C=O), 1611, 1482 (Ar), 825 (CF_3); HRMS (ESI): *m/z* calcd for $C_{17}H_9ClF_3O_5$ $[M-H]^+$: 385.0091; found: 384.9819.

***1,7-Dihydroxy-3-methyl-7-(trifluoromethyl)-6a,12b-dihydro-6H,7H-chromeno[3,4-c]chromen-6-one* (3p):**

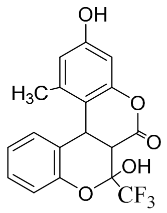

^{1}H-NMR ($CDCl_3$, 400 MHz) δ: 2.42 (s, 3H, CH_3), 3.53 (d, J = 4 Hz, 1H, CH), 4.58 (d, J = 4 Hz, 1H, CH), 5.02 (s, 1H, -OH), 6.46 (s, 1H, -OH), 6.59 (dt, J_1 = 8 Hz, J_2 = 0.8 Hz, 1H, Ar-H), 6.67 (d, J = 2.4 Hz, 1H, Ar-H), 6.86 (td, J_1 = 4 Hz, J_2 = 0.8 Hz, 1H, Ar-H), 7.03 (dd, J_1 = 8 Hz, J_2 = 0.8 Hz, 1H, Ar-H), 7.21 (d, J = 4 Hz, 1H, Ar-H), 7.43 (d, J = 0.8 Hz, 1H, Ar-H); ^{13}C-NMR (100 MHz, $CDCl_3$) δ: 18.95 (-CH_3), 31.20 (C3), 39.83 (C2), 95.82 (q, J = 26 Hz, C16), 102.53 (C12), 113.25 (C8), 114.46 (C6), 117.66 (C4), 118.43 (C14), 122.02 (q, J = 229 Hz, C17), 122.66 (C13), 126.75 (C15), 129.86 (C10), 138.93 (C7), 150.35 (C5), 152.57 (C11), 156.21 (C9), 168.39 (C1); IR (KBr) $_{vmax}$ (cm^{-1}): 3461 (OH), 1706 (C=O), 1633, 1594 (Ar), 753 (CF_3); HRMS (ESI): *m/z* calcd for $C_{18}H_{12}F_3O_5$ $[M-H]^+$: 365.0637; found: 365.0378.

Figure S2-S25:1H NMR, 13C NMR, and HRMS spectra of compound 3a-p.

^{1}H and ^{13}C NMR spectra

S2: 3a

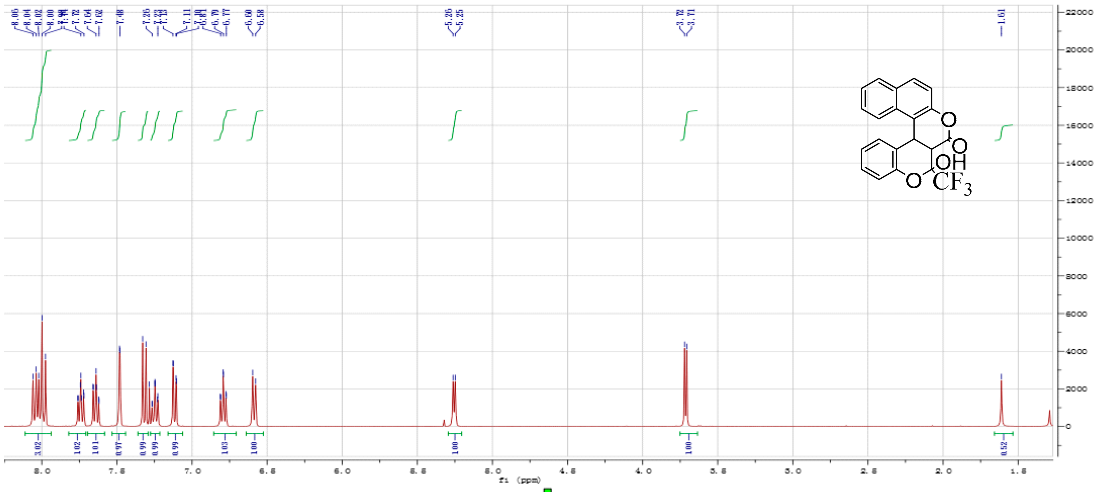

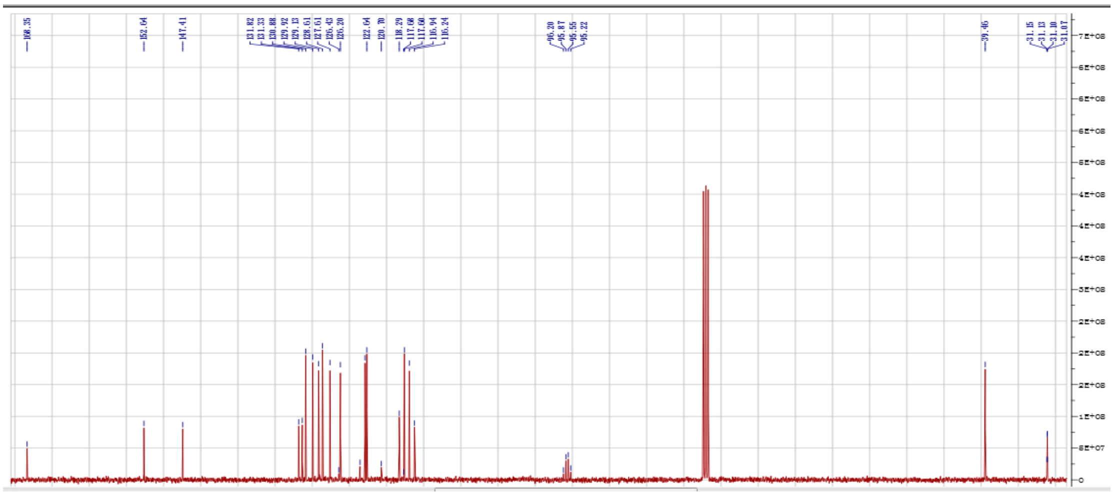

3a HRMS (ESI): *m/z* calcd for $C_{21}H_{12}F_3O_4$ $[M - H]^+$: 385.0688; found: 385.0688.

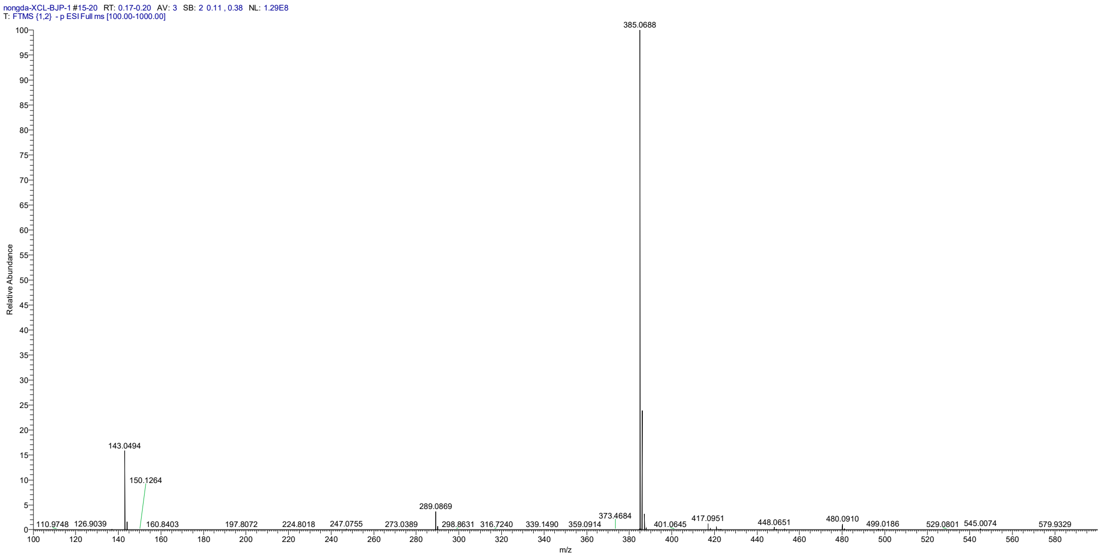

S3:3b

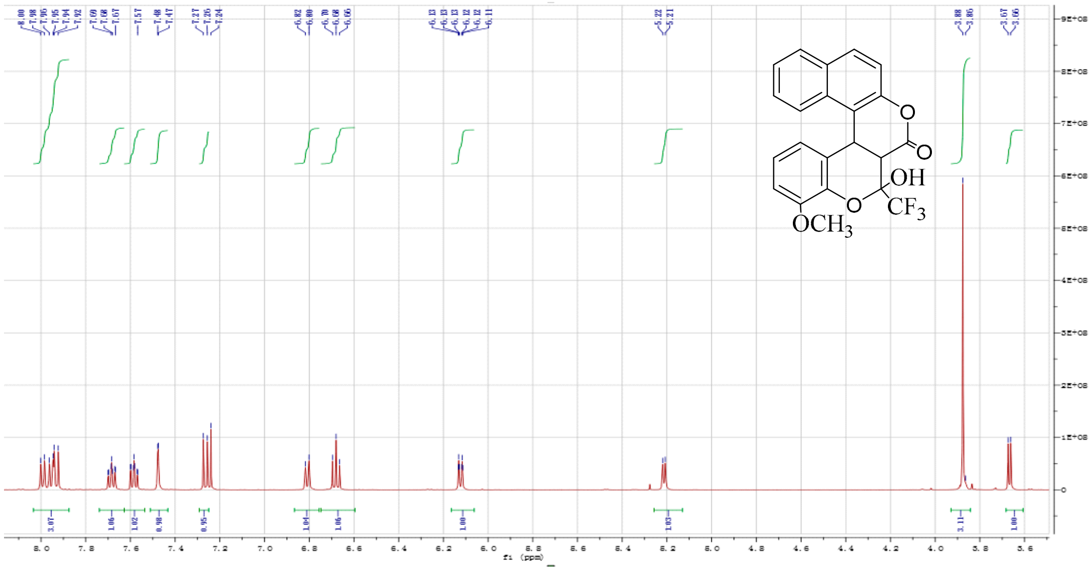

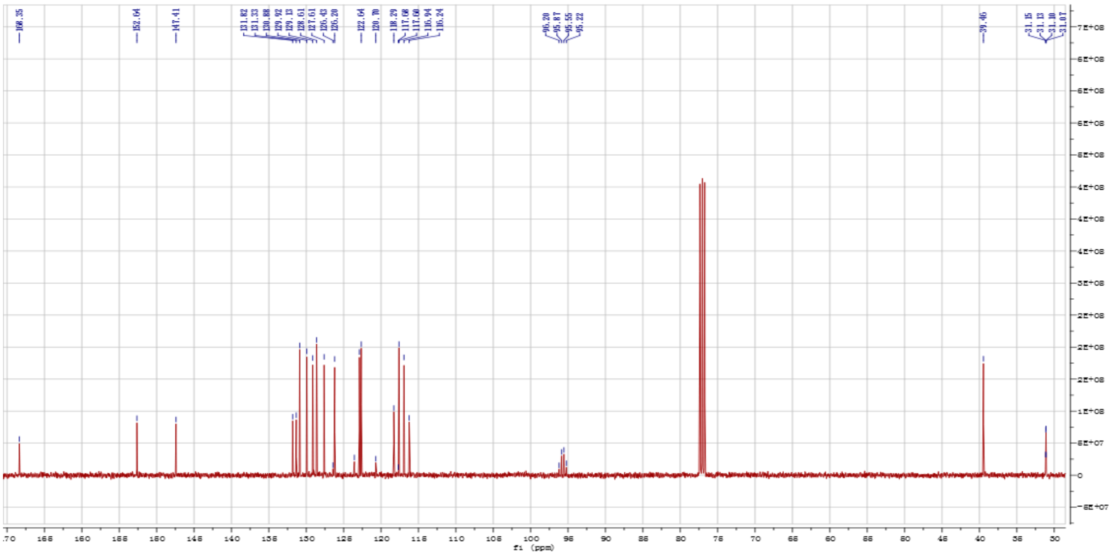

3b HRMS (ESI): *m/z* calcd for $C_{22}H_{15}F_3O_5$ [M - H]$^+$: 415.0793; found: 415.0795.

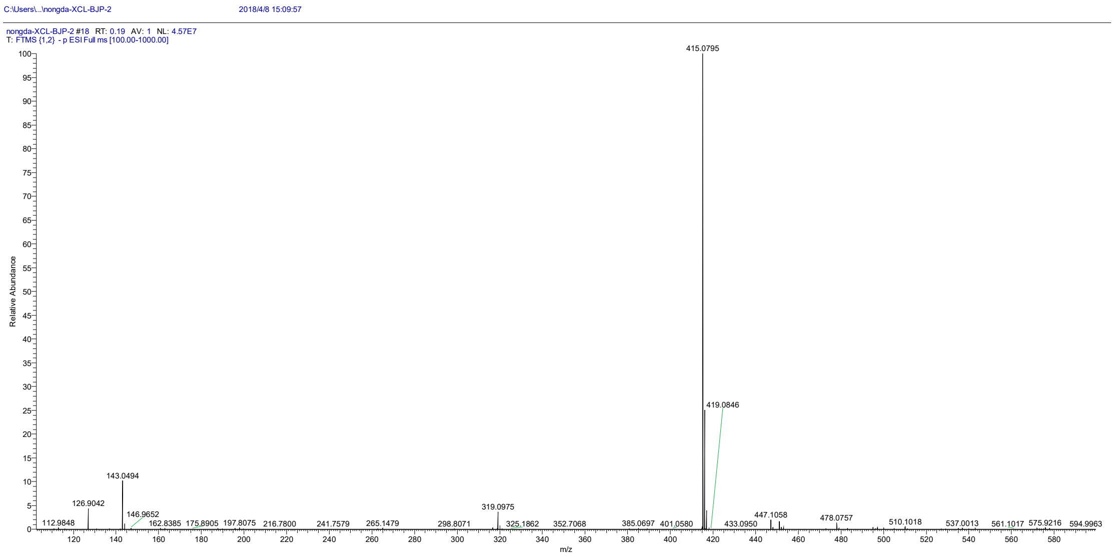

S4:**3c**

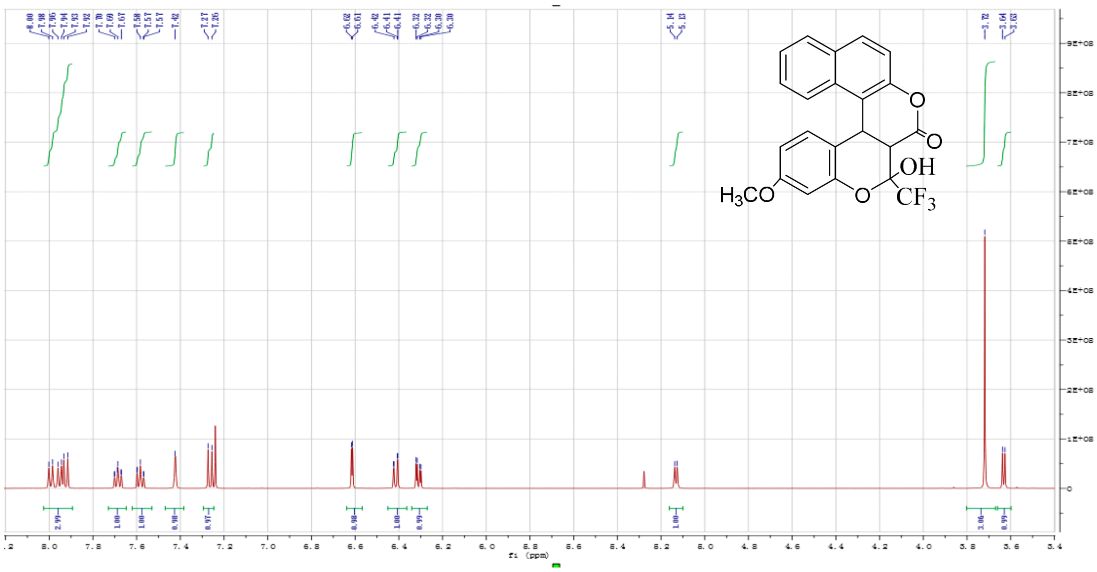

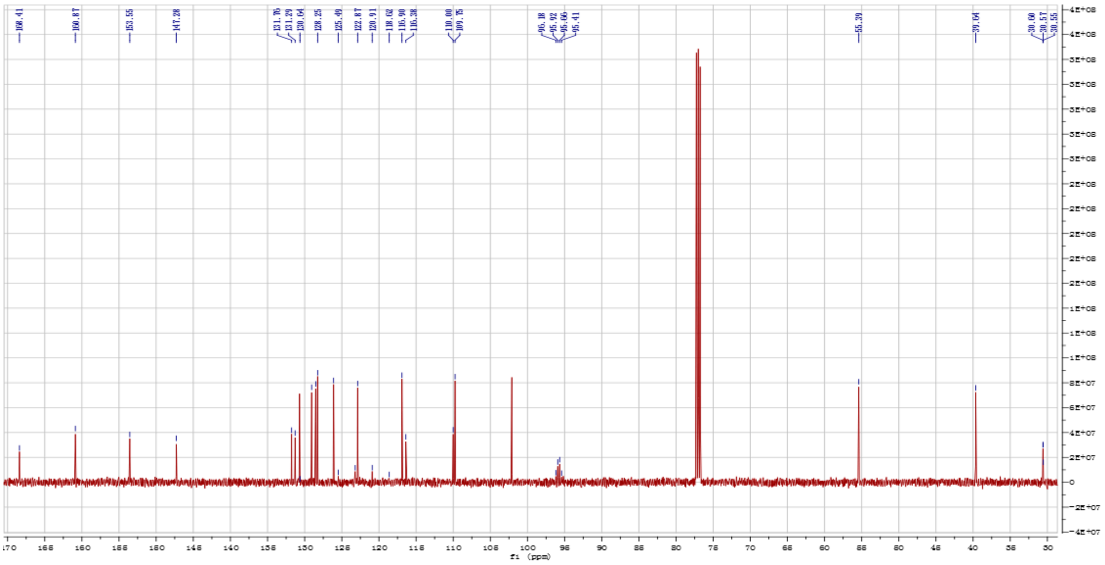

3c HRMS (ESI): *m/z* calcd for $C_{22}H_{15}F_3O_5$ $[M - H]^+$: 415.0793; found: 415.0792.

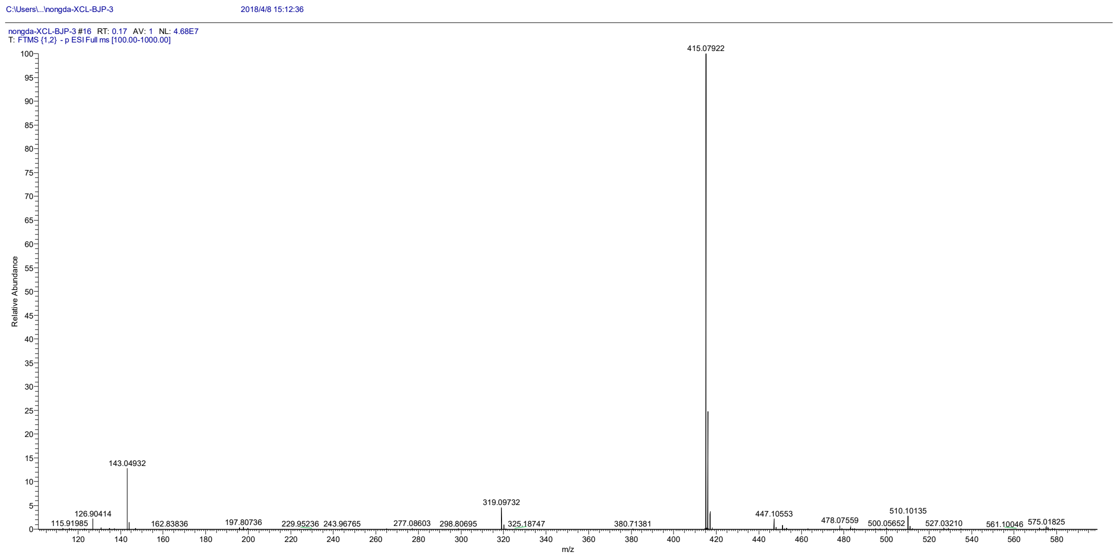

S5:3d

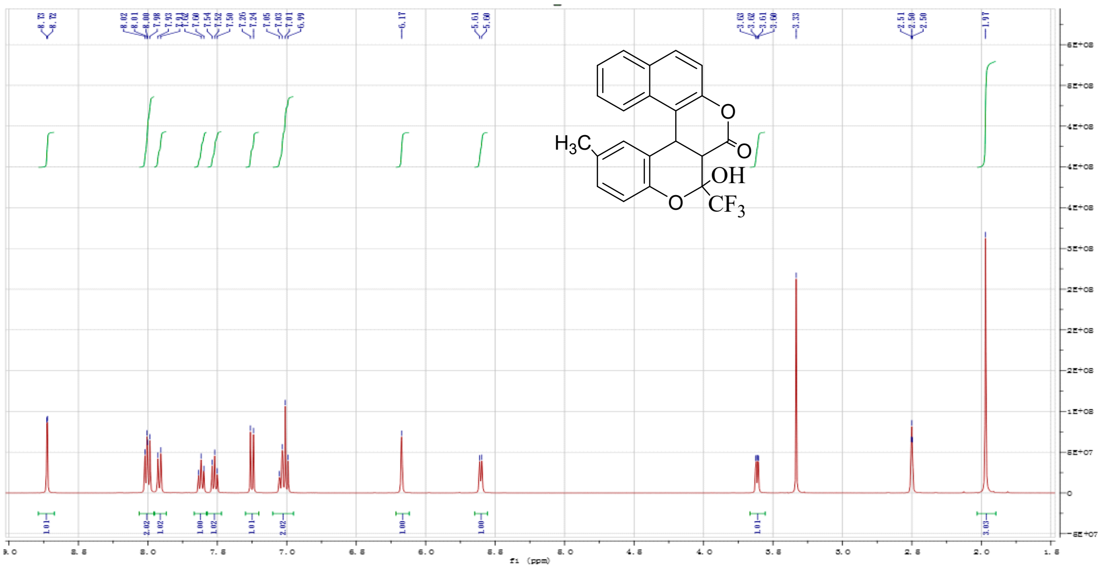

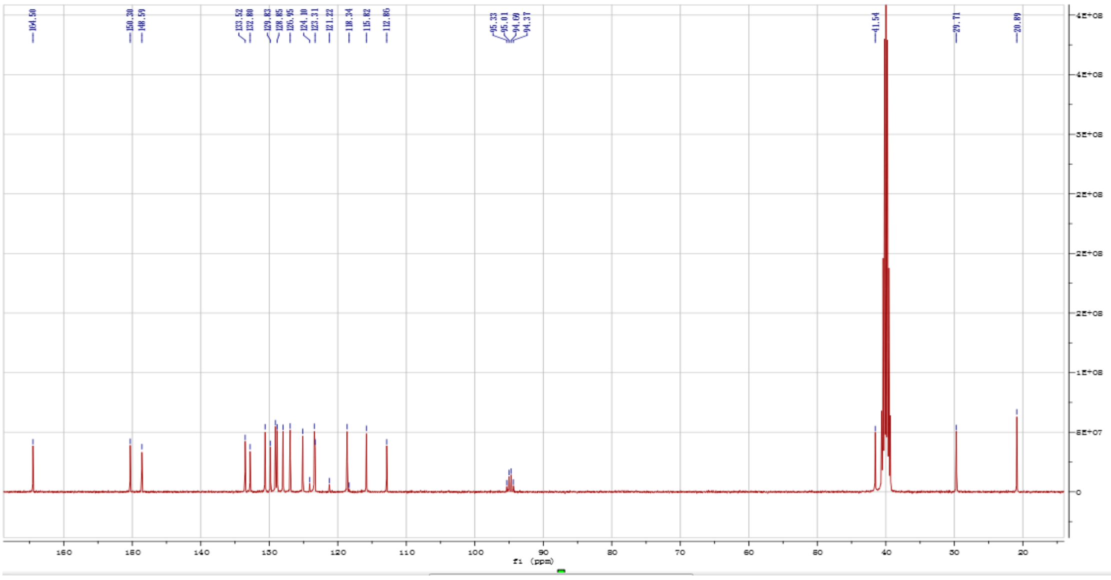

3d HRMS (ESI): *m/z* calcd for $C_{22}H_{15}F_3O_4$ $[M - H]^+$: 399.0844; found: 399.0805.

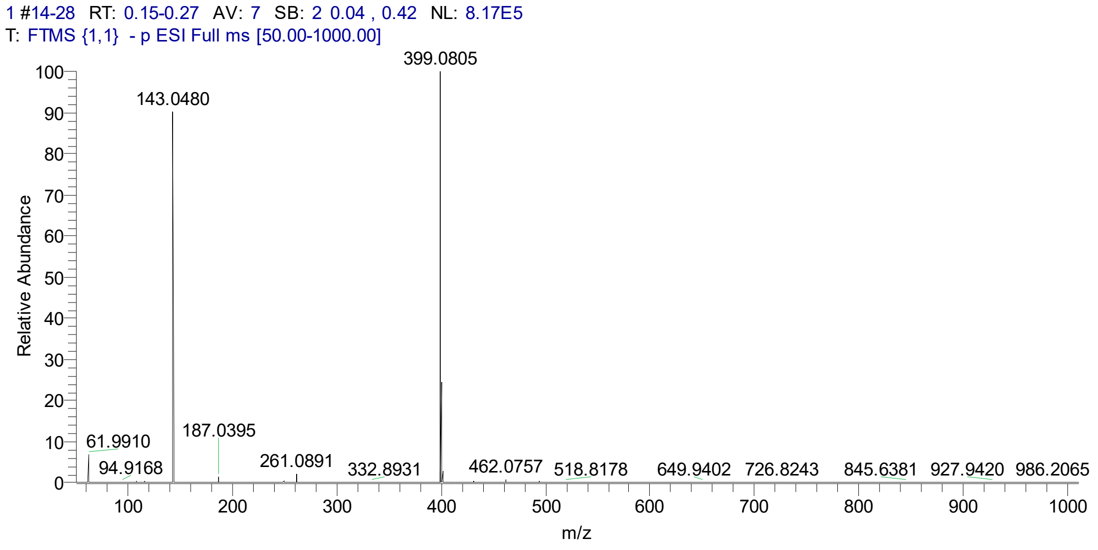

S6:3e

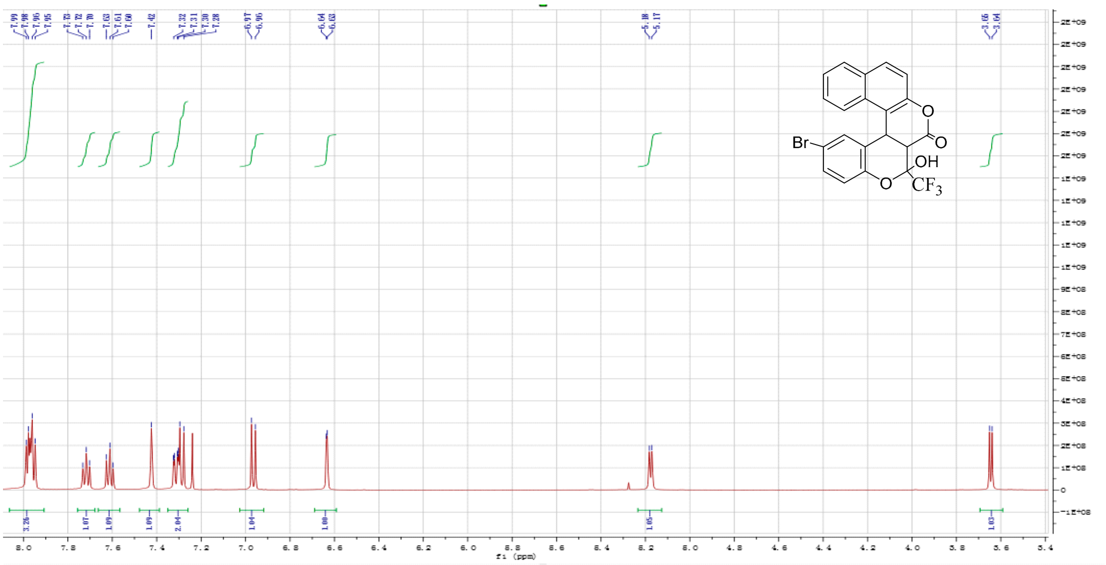

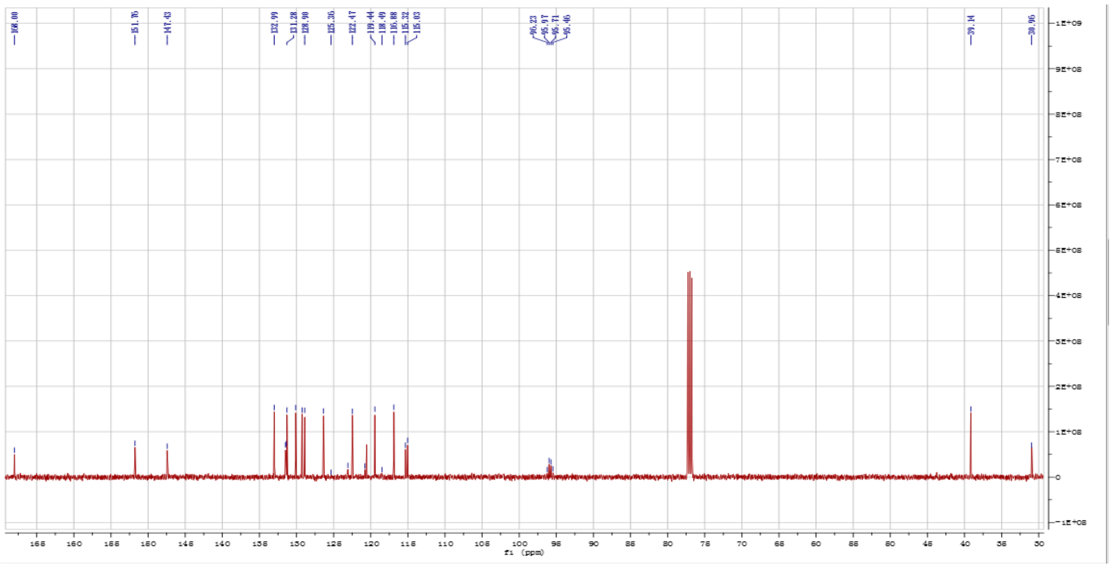

3e HRMS (ESI): *m/z* calcd for $C_{21}H_{11}BrF_3O_4$ $[M - H]^+$: 462.9793; found: 462.9793.

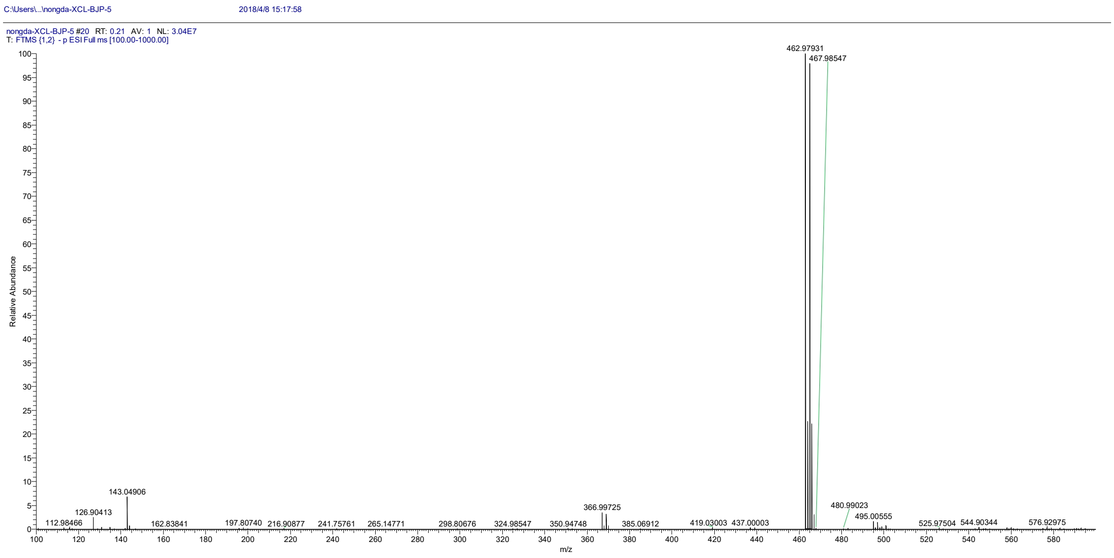

S7: 3f

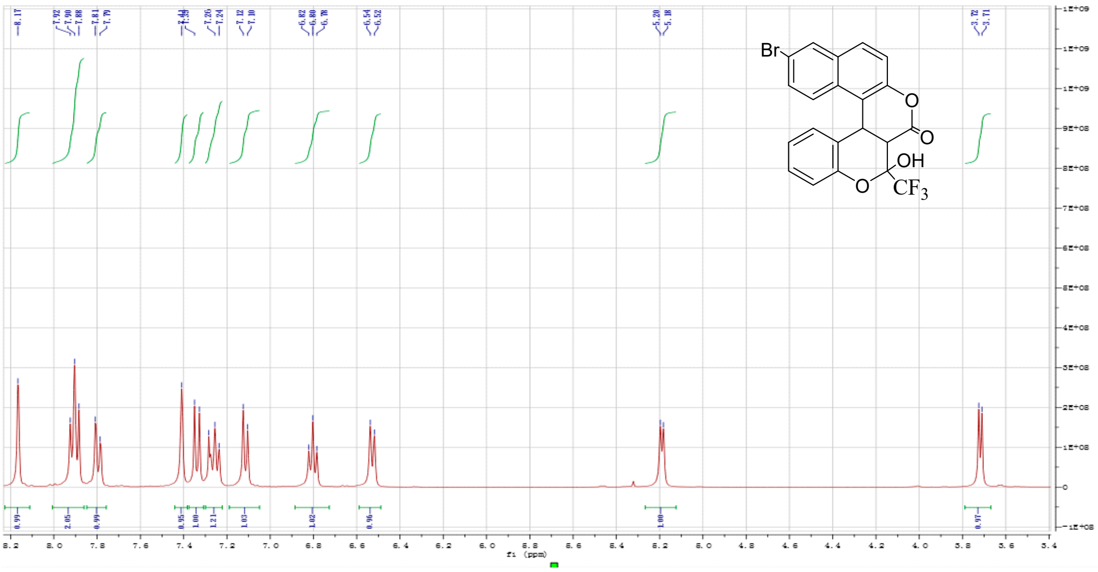

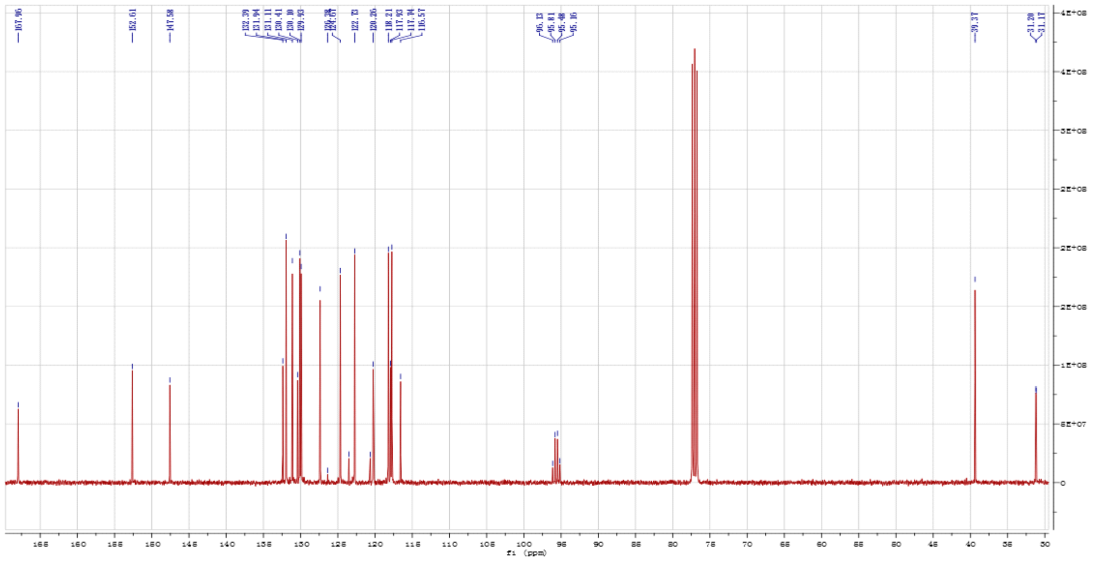

3f HRMS (ESI): *m/z* calcd for $C_{21}H_{11}BrF_3O_4$ $[M - H]^+$: 462.9793; found: 462.9791.

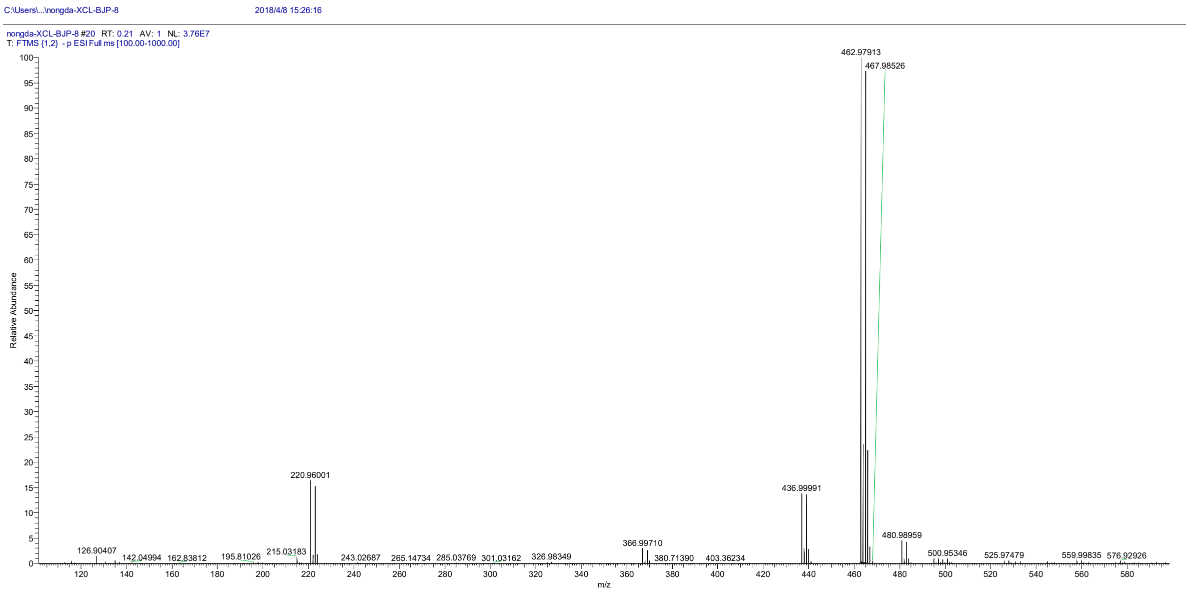

S8: 3g

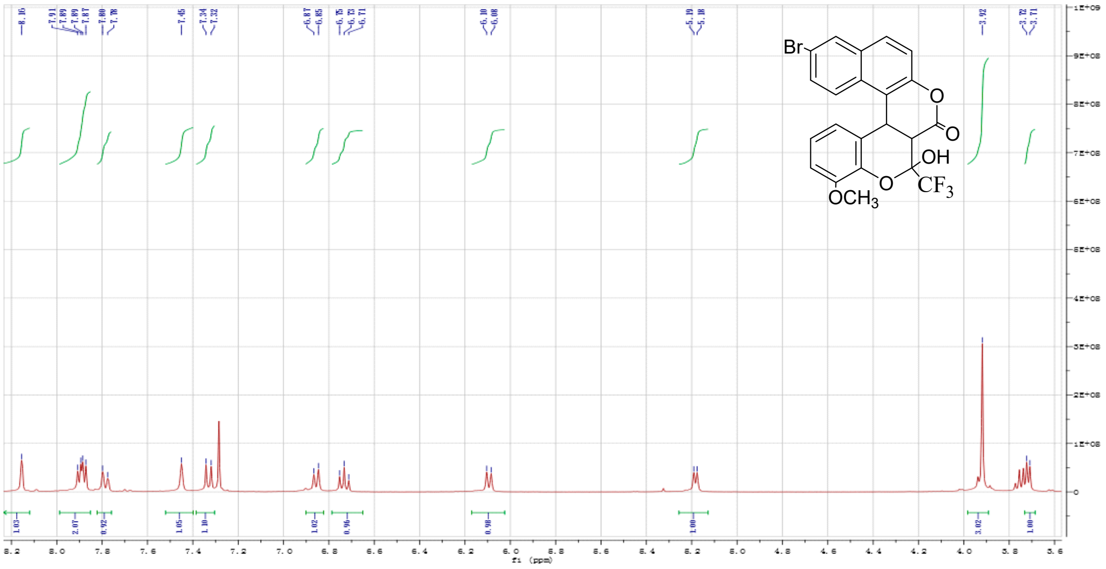

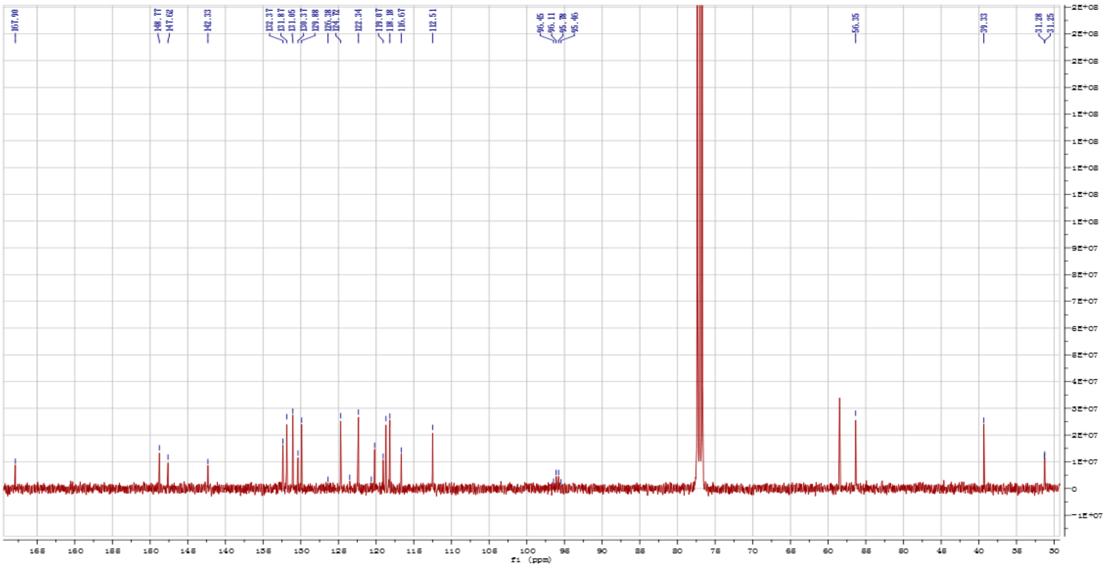

3g HRMS (ESI): *m/z* calcd for $C_{22}H_{14}BrF_3O_5$ $[M - H]^+$: 492.9898; found: 492.9850.

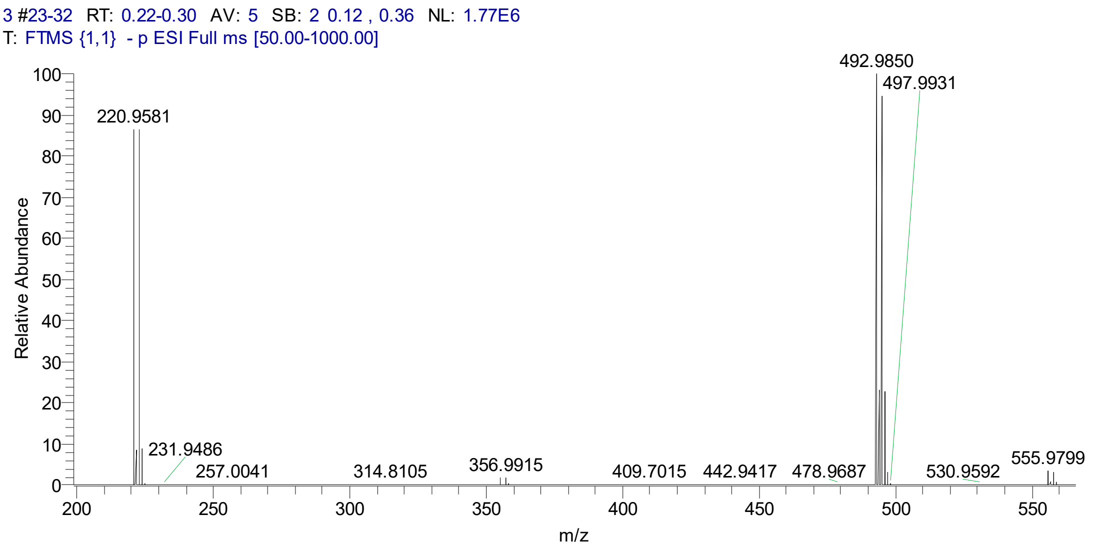

S9: 3h

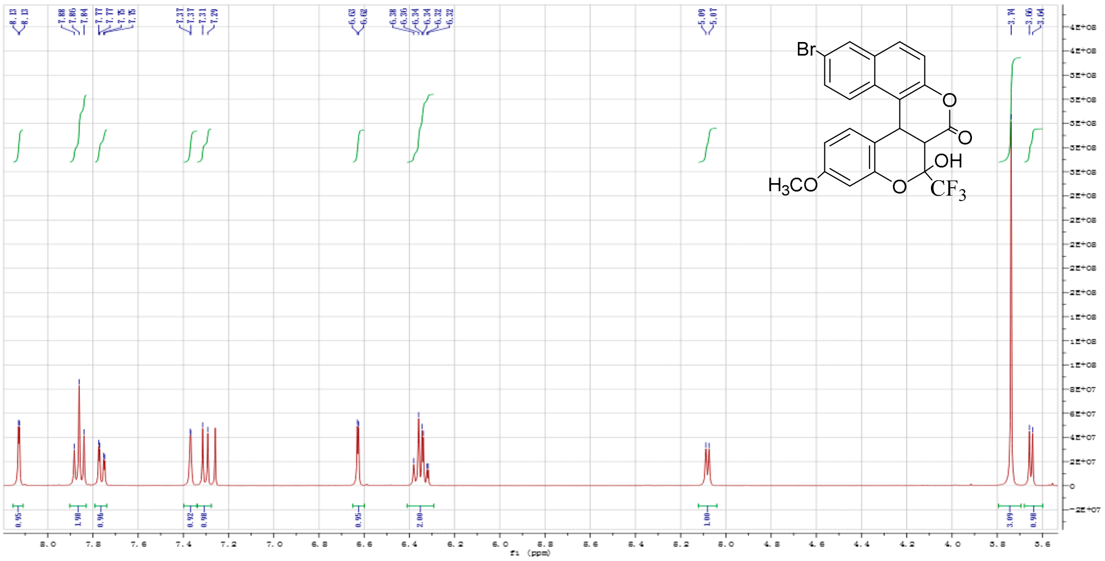

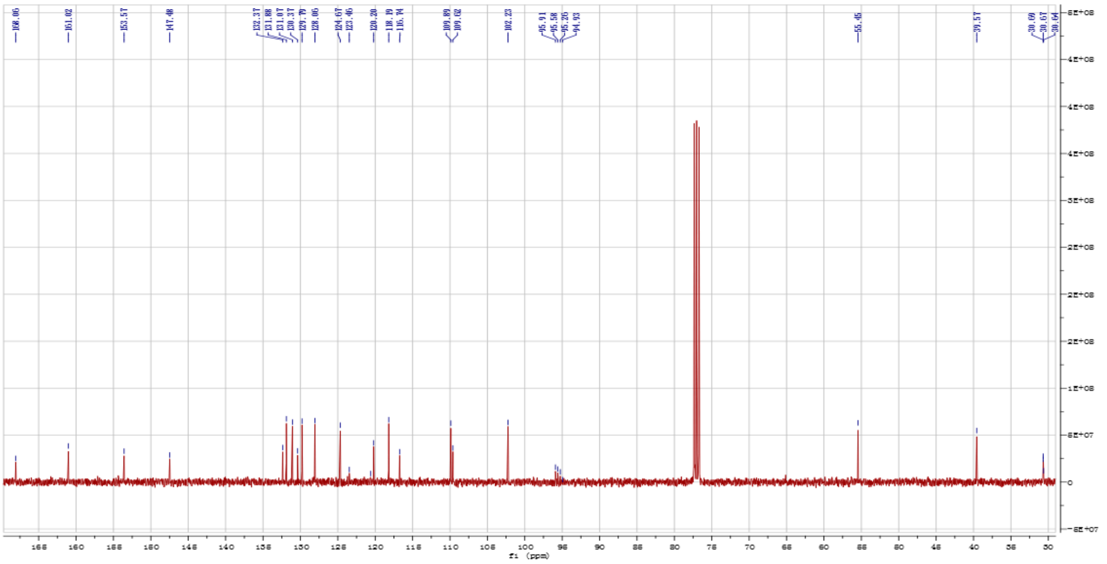

3h HRMS (ESI): *m/z* calcd for $C_{22}H_{14}BrF_3O_5$ $[M - H]^+$: 492.9898; found: 492.9510.

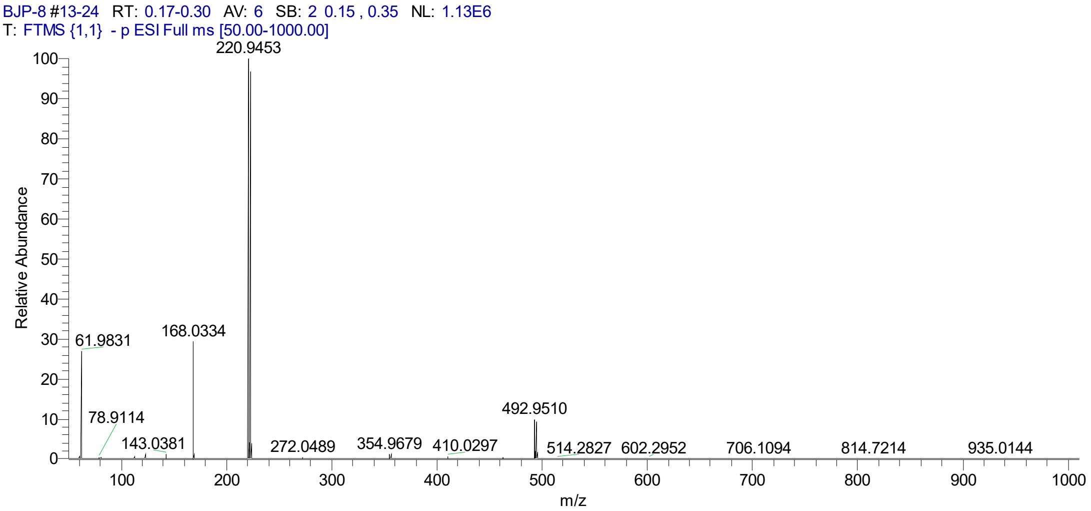

S10: 3i

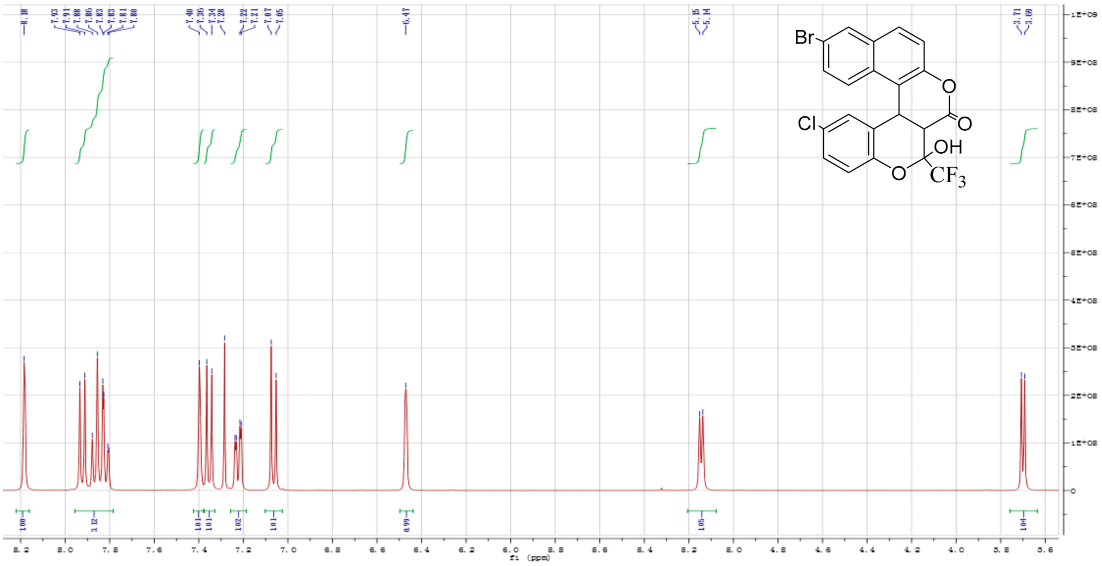

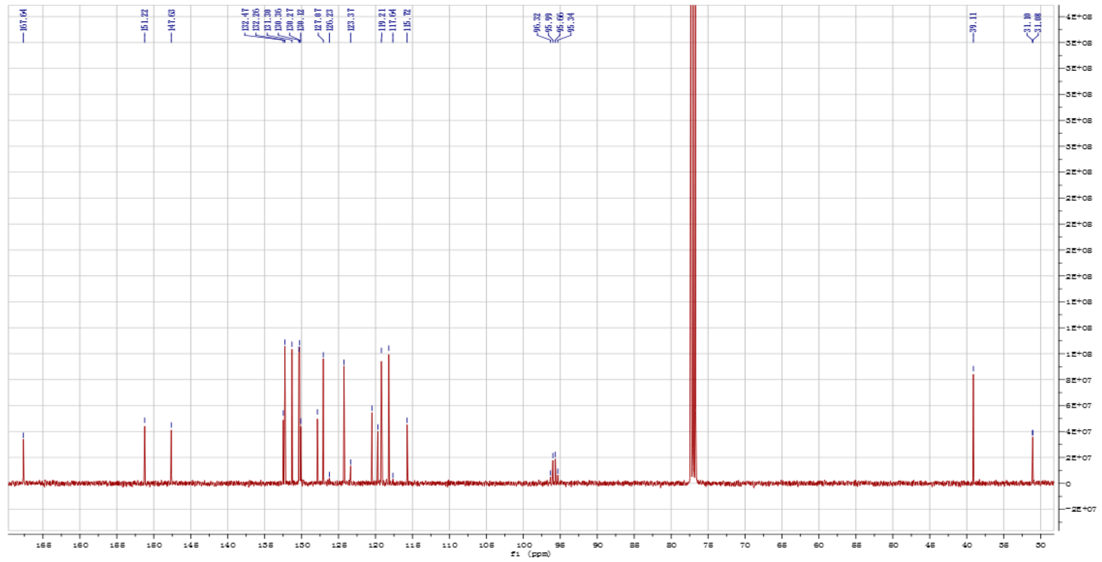

3i HRMS (ESI): *m/z* calcd for $C_{21}H_{11}BrClF_3O_4$ $[M + H]^+$: 498.9560; found: 498.9331.

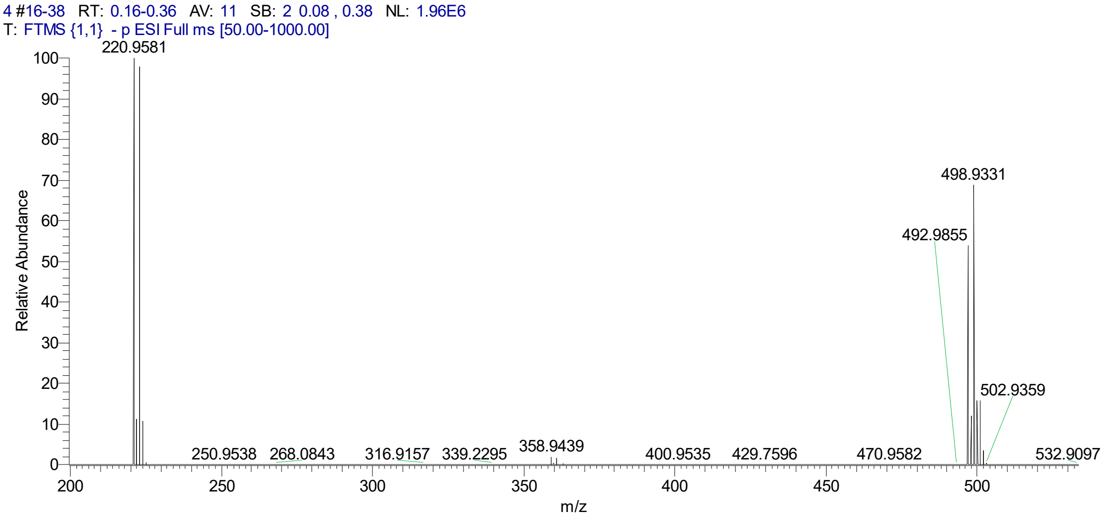

S11: 3j

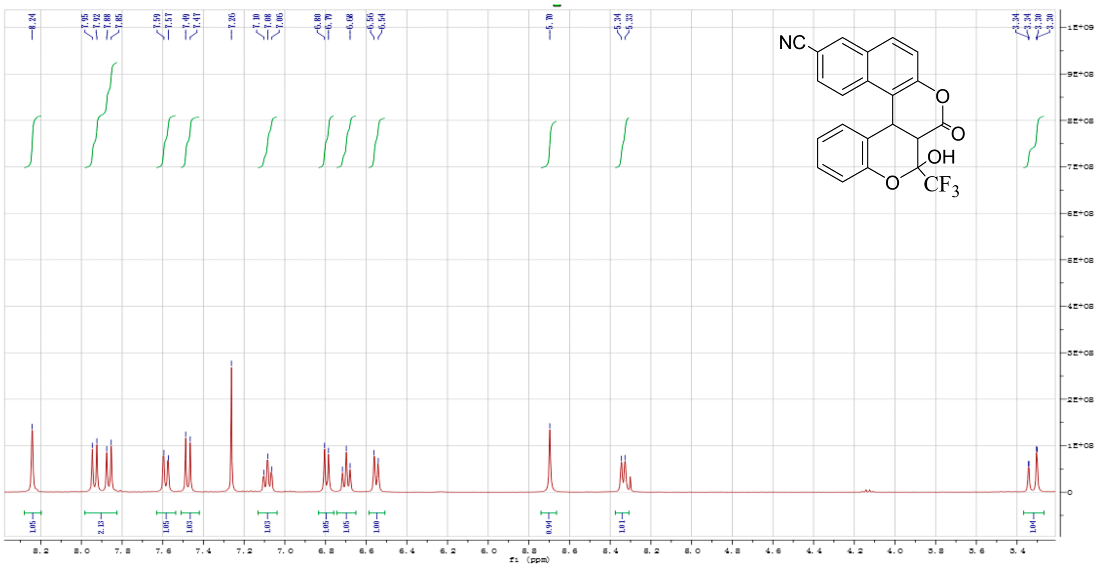

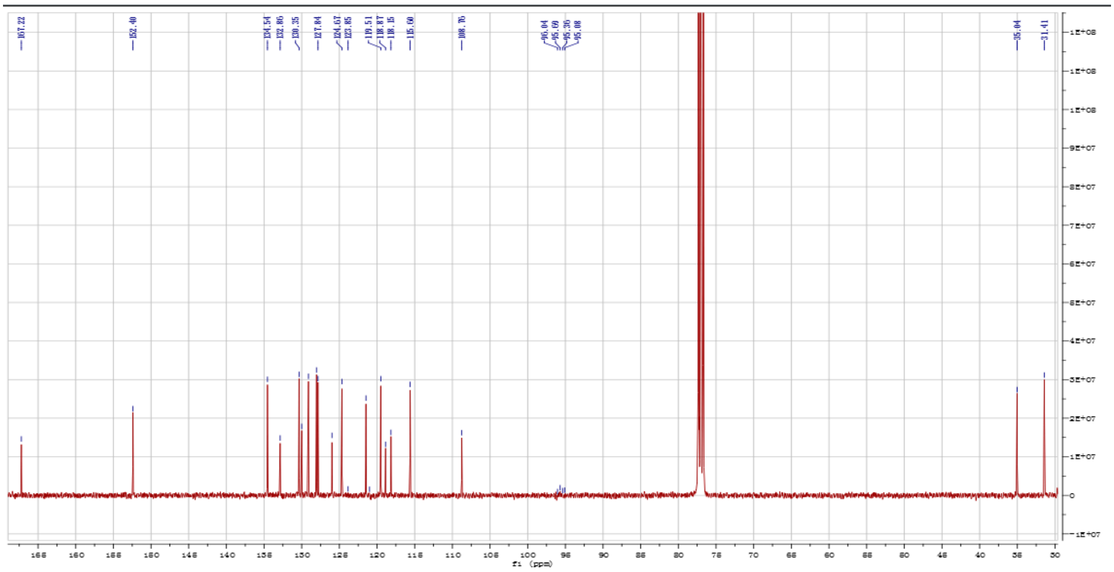

3j HRMS (ESI): *m/z* calcd for $C_{22}H_{12}F_3NO_4$ $[M - H]^+$: 410.0640; found: 410.0600.

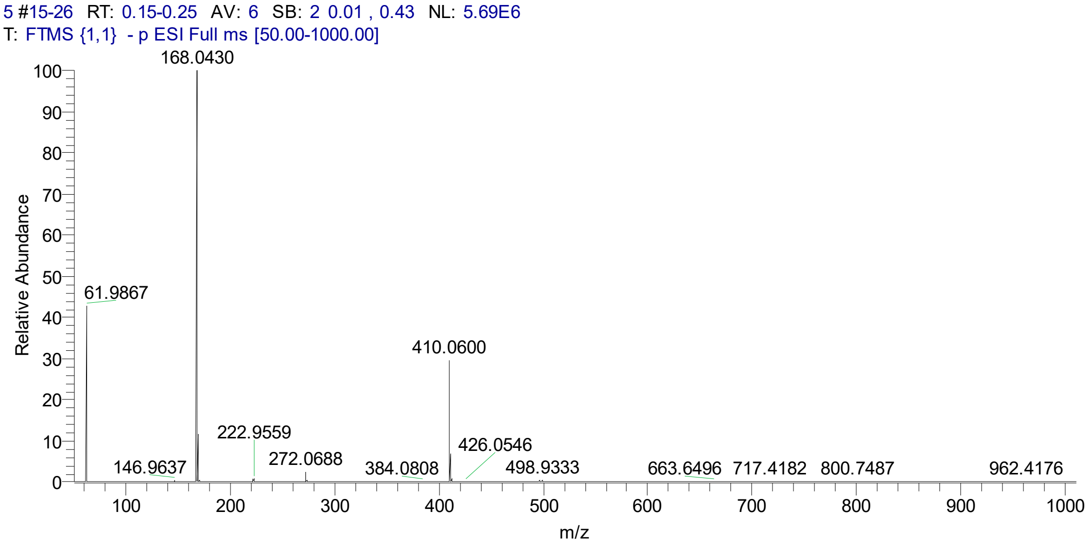

S12: 3k

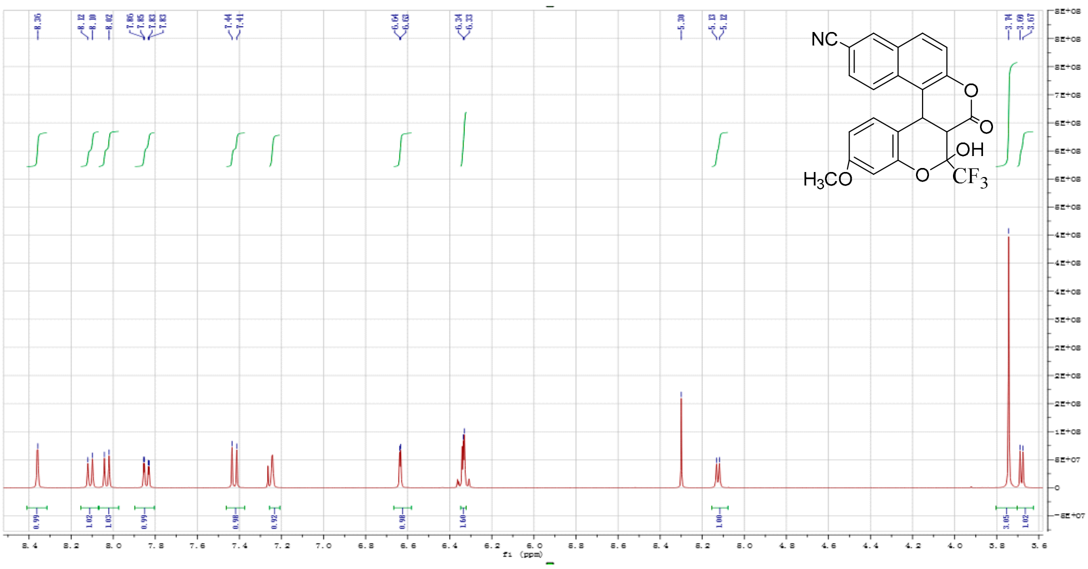

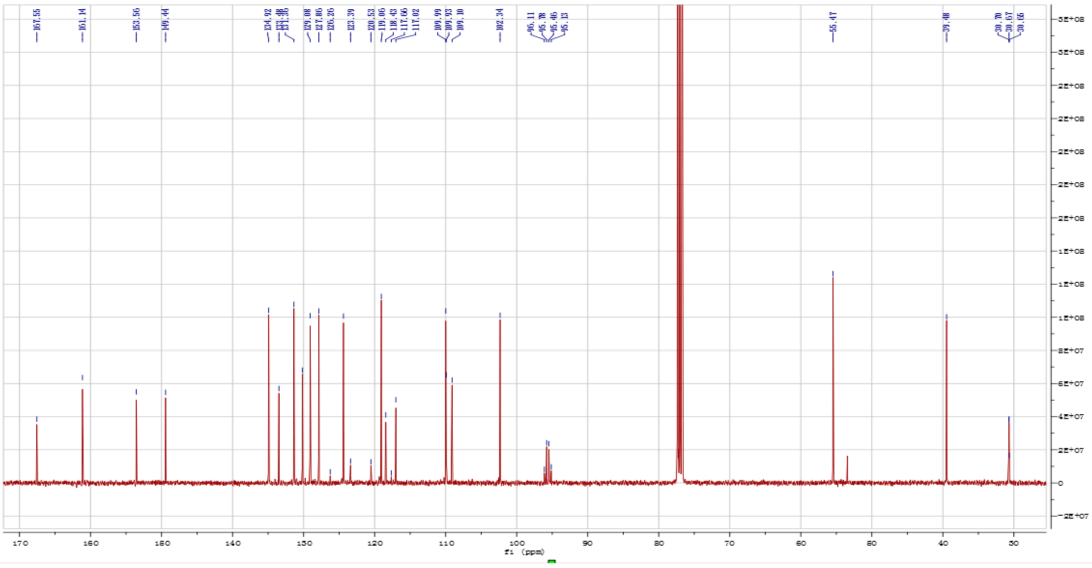

3k HRMS (ESI): *m/z* calcd for $C_{23}H_{13}F_3NO_4$ [M - H]$^+$: 440.0746; found: 440.0743.

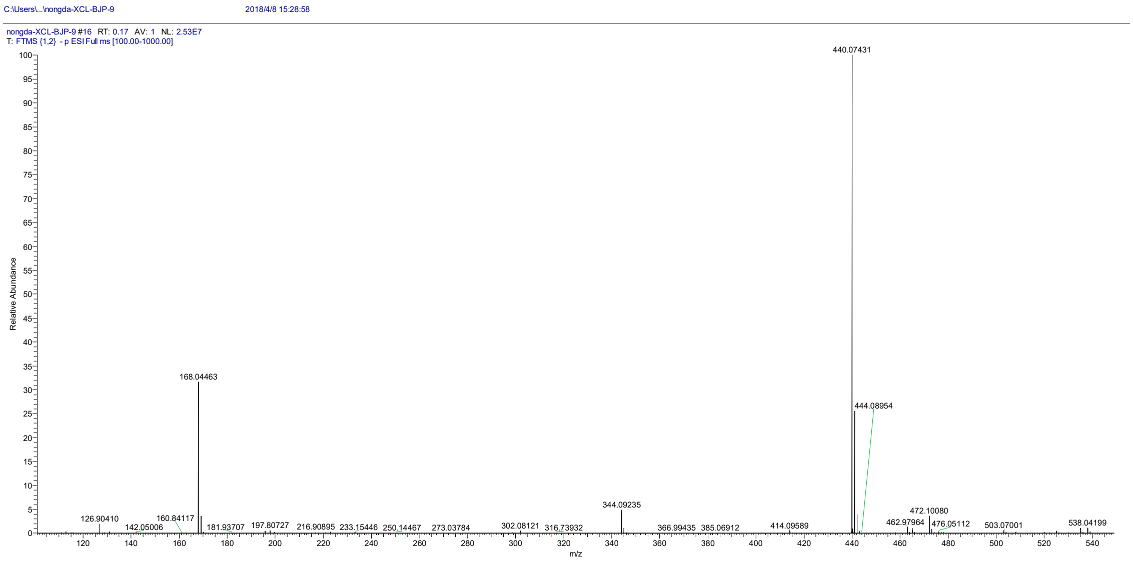

S13: 3l

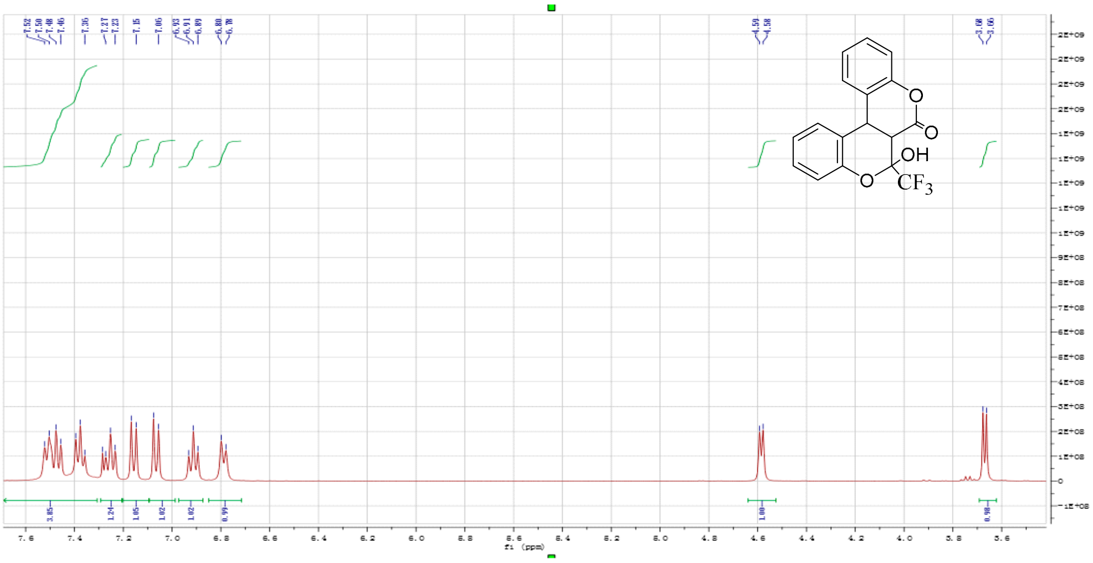

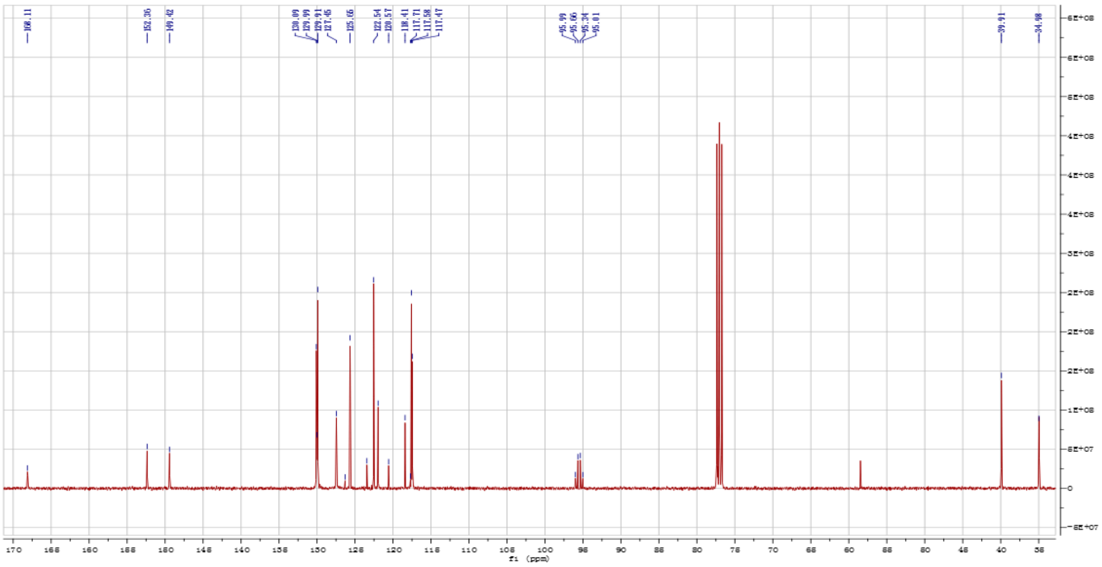

3l HRMS (ESI): *m/z* calcd for $C_{17}H_{10}F_3O_4$ $[M - H]^+$: 335.0531; found: 335.0298.

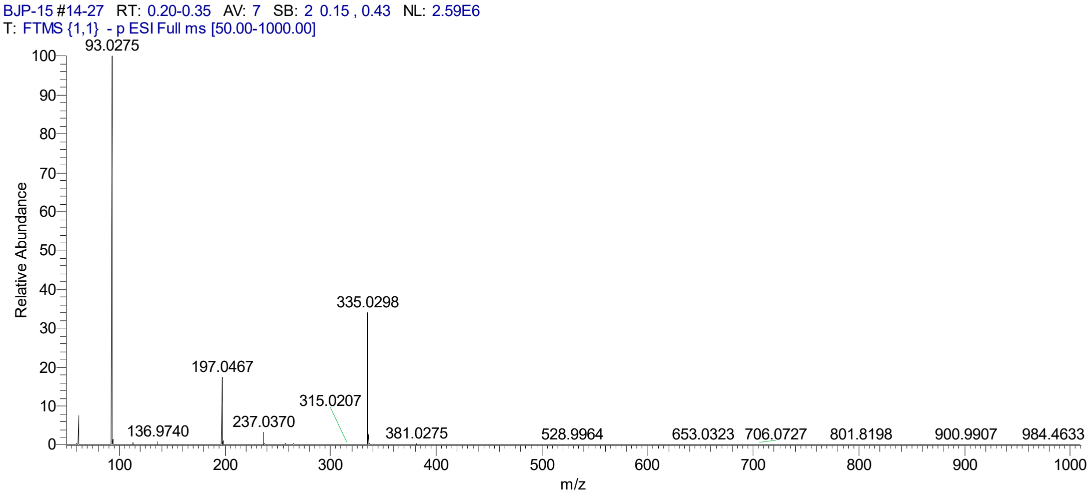

S14: 3m

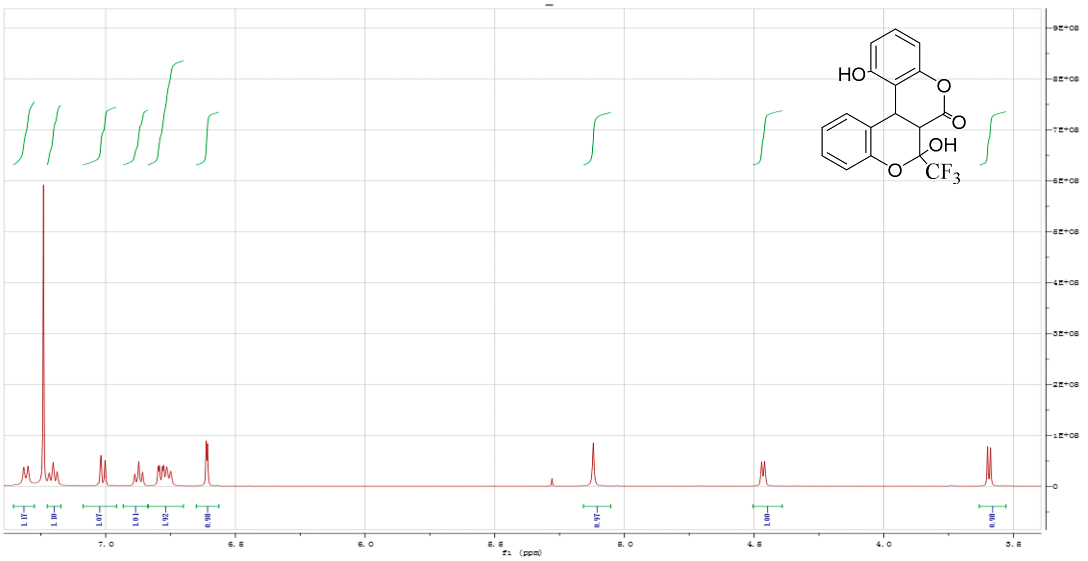

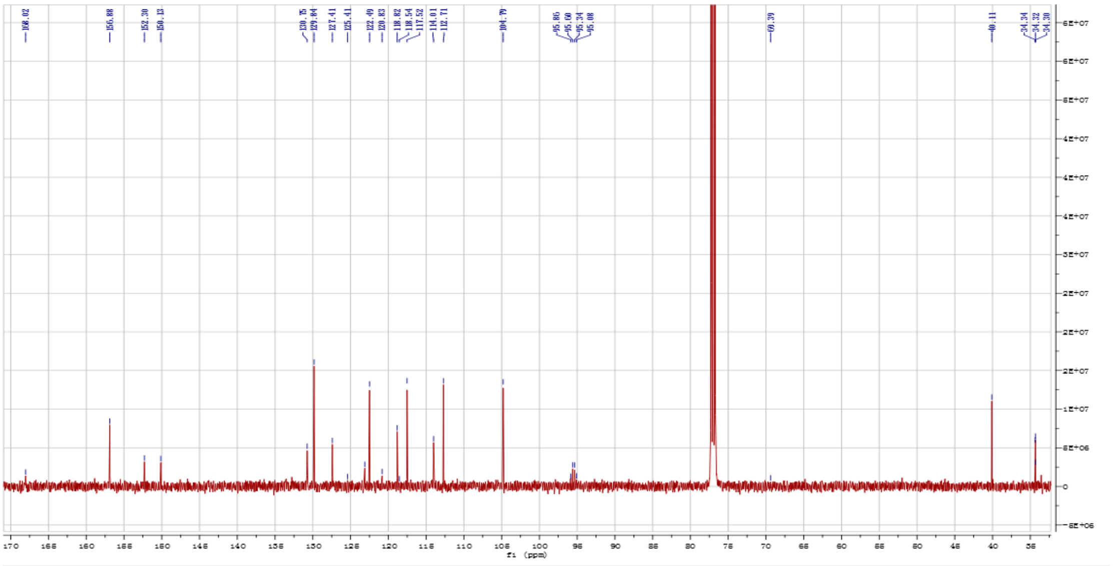

3m HRMS (ESI): *m/z* calcd for $C_{17}H_{10}F_{3}O_{5}$ $[M - H]^{+}$: 351.0480; found: 351.0236.

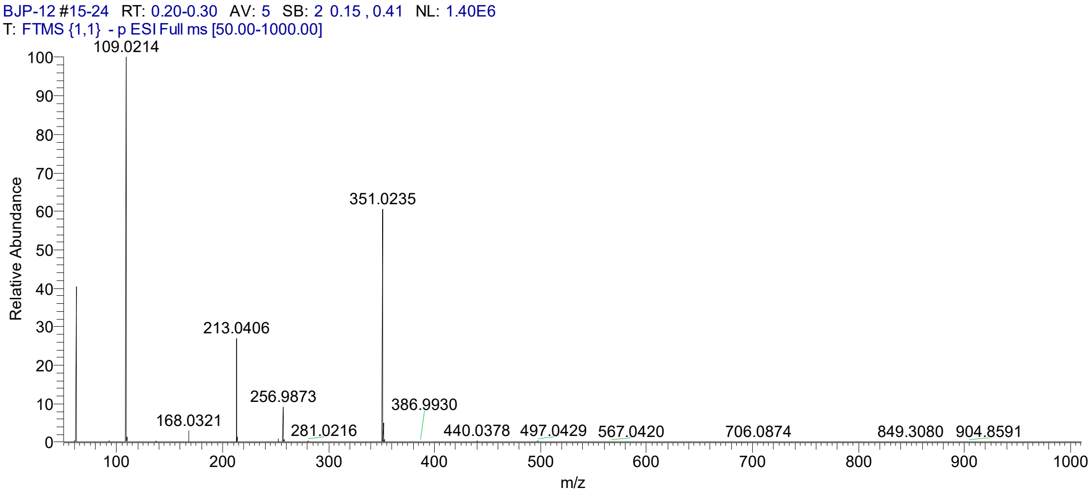

S15: 3n

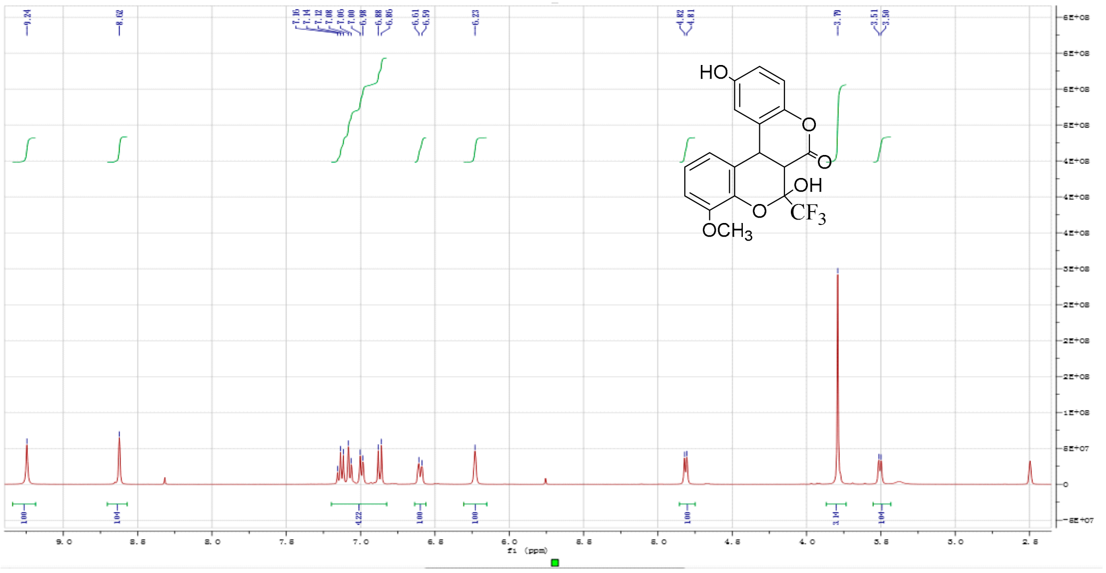

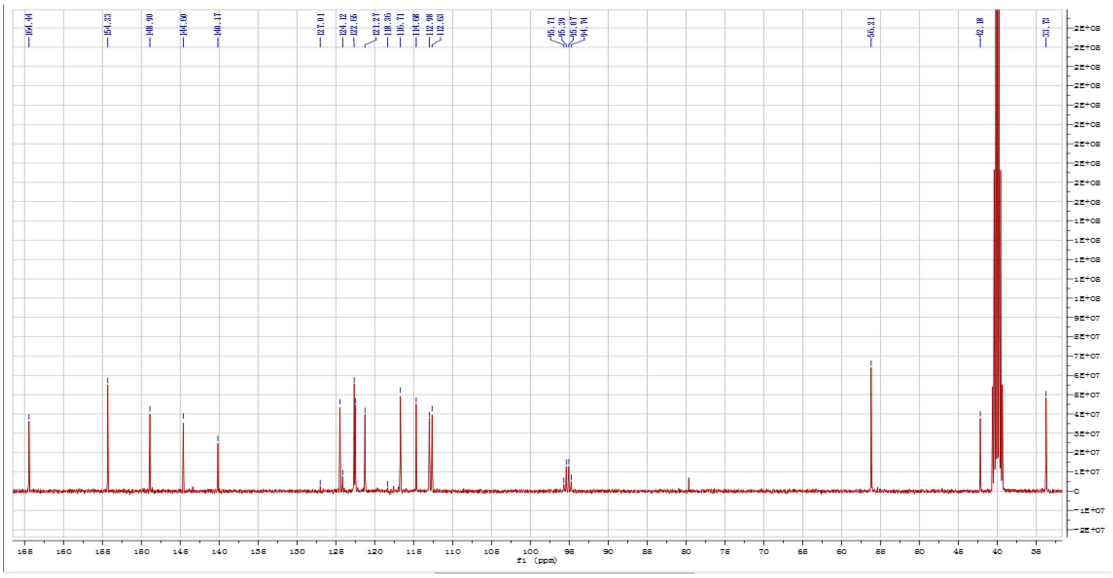

3n HRMS (ESI): *m/z* calcd for $C_{18}H_{12}F_3O_6$ $[M - H]^+$: 381.0586; found: 381.0317.

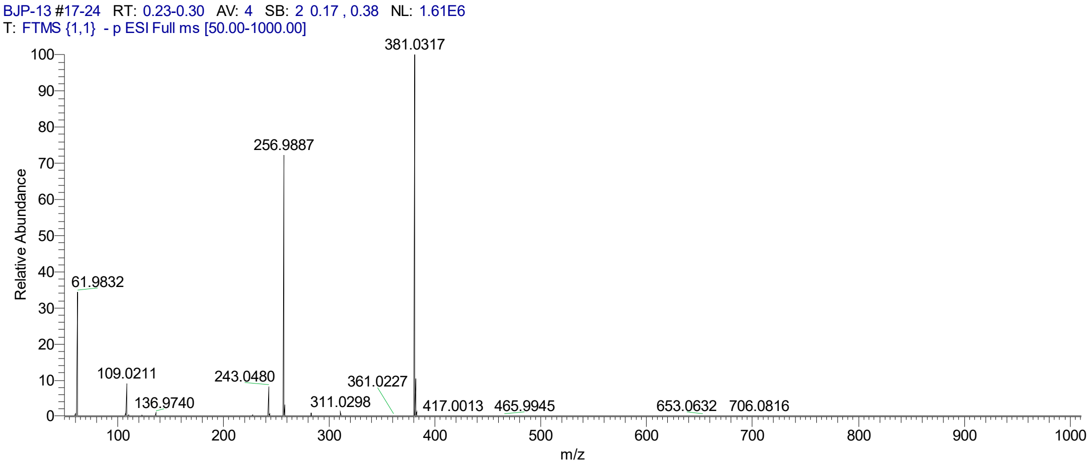

S16: 3o

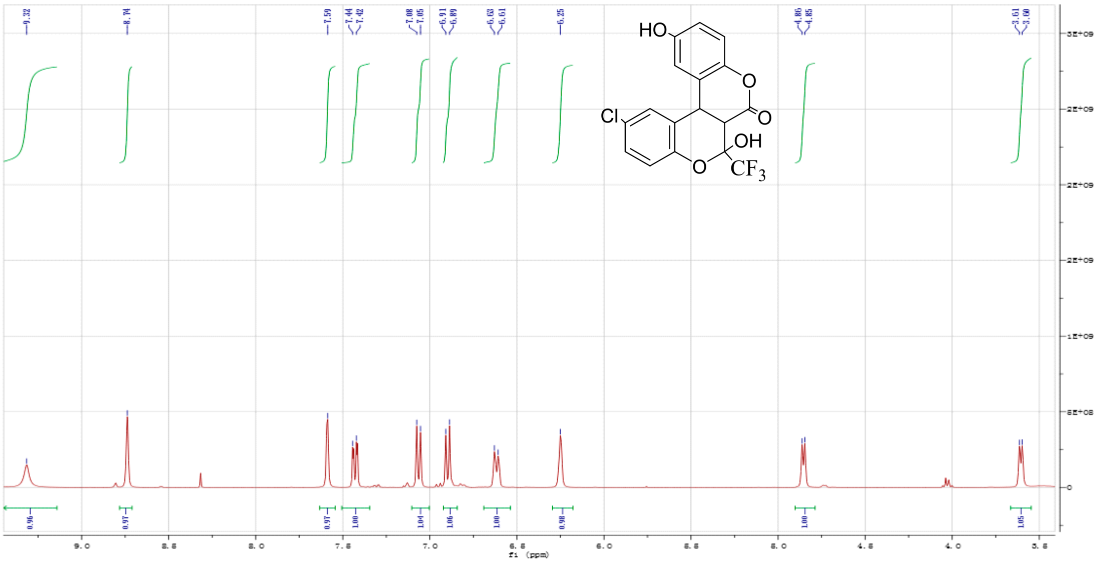

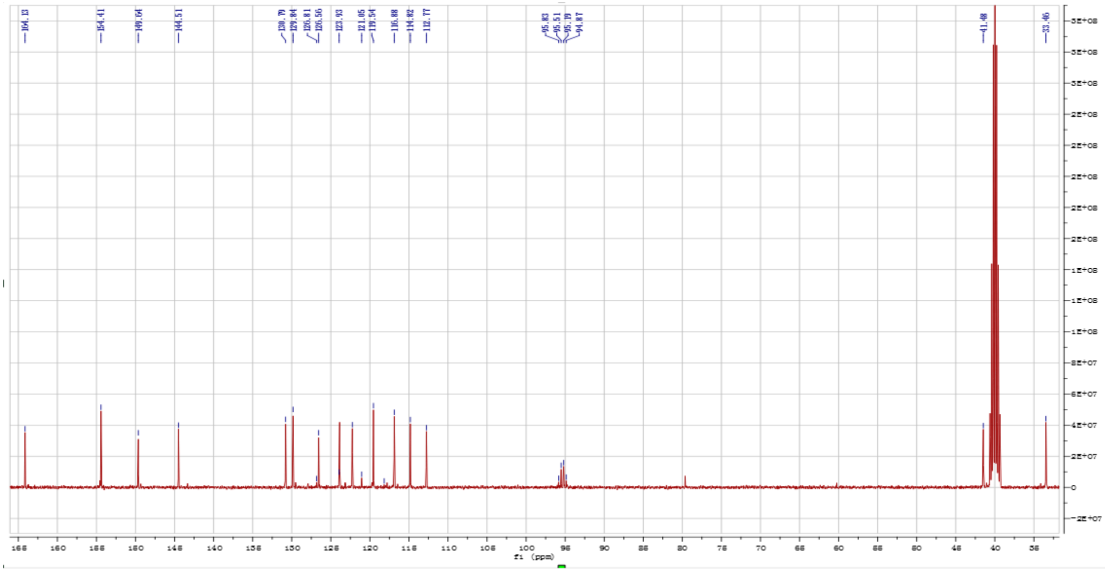

3o HRMS (ESI): *m/z* calcd for $C_{17}H_9ClF_3O_5$ $[M - H]^+$: 385.0091; found: 384.9819.

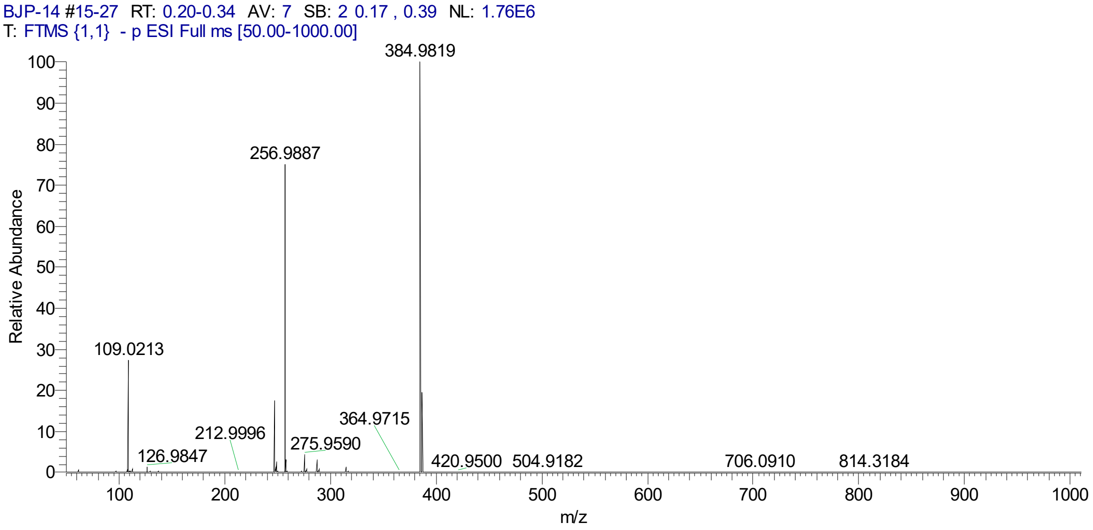

S17: 3p

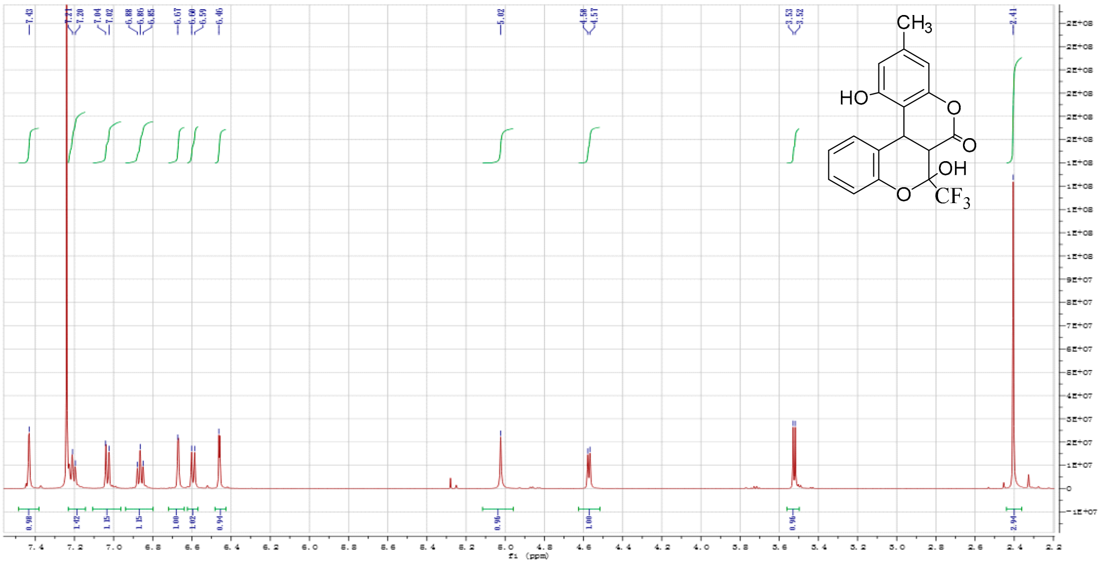

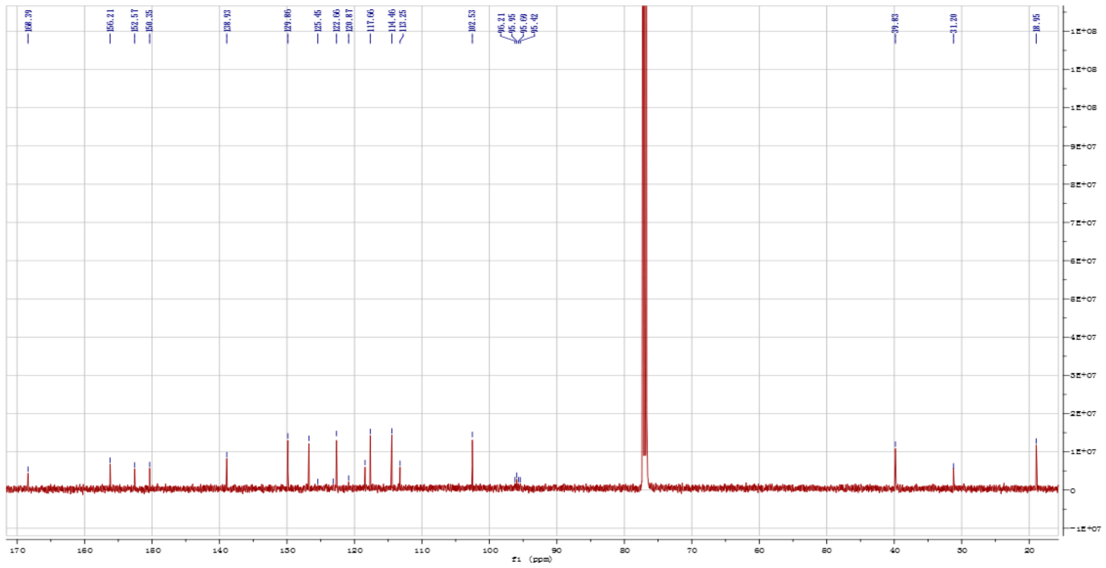

3p HRMS (ESI): *m/z* calcd for $C_{18}H_{12}F_3O_5$ $[M - H]^+$: 365.0637; found: 365.0378.

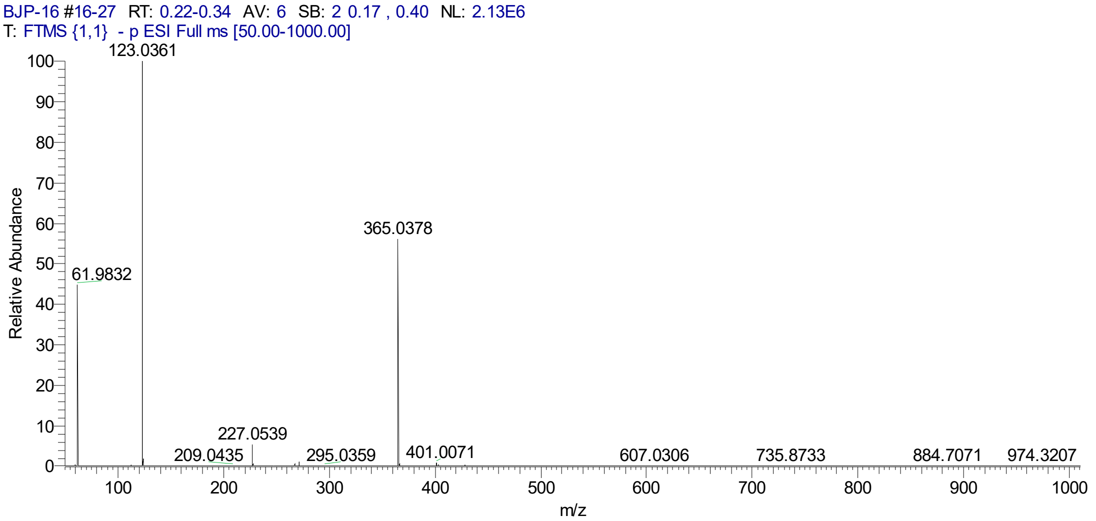

Supplement: Supplementary file 1 [file molecules-24-01745-s001.pdf]
